# Comparative Structure and Function Analysis of the RIG-I-Like Receptors: RIG-I and MDA5

**DOI:** 10.3389/fimmu.2019.01586

**Published:** 2019-07-17

**Authors:** Morgan Brisse, Hinh Ly

**Affiliations:** ^1^Biochemistry, Molecular Biology, and Biophysics Graduate Program, University of Minnesota, Twin Cities, St. Paul, MN, United States; ^2^Department of Veterinary & Biomedical Sciences, University of Minnesota, Twin Cities, St. Paul, MN, United States

**Keywords:** RIG-I, MDA5, PAMP, CARD, interferon, antiviral, inflammation, PRRs

## Abstract

RIG-I (Retinoic acid-inducible gene I) and MDA5 (Melanoma Differentiation-Associated protein 5), collectively known as the RIG-I-like receptors (RLRs), are key protein sensors of the pathogen-associated molecular patterns (PAMPs) in the form of viral double-stranded RNA (dsRNA) motifs to induce expression of type 1 interferons (IFN1) (IFNα and IFNβ) and other pro-inflammatory cytokines during the early stage of viral infection. While RIG-I and MDA5 share many genetic, structural and functional similarities, there is increasing evidence that they can have significantly different strategies to recognize different pathogens, PAMPs, and in different host species. This review article discusses the similarities and differences between RIG-I and MDA5 from multiple perspectives, including their structures, evolution and functional relationships with other cellular proteins, their differential mechanisms of distinguishing between host and viral dsRNAs and interactions with host and viral protein factors, and their immunogenic signaling. A comprehensive comparative analysis can help inform future studies of RIG-I and MDA5 in order to fully understand their functions in order to optimize potential therapeutic approaches targeting them.

## Introduction

RIG-I (Retinoic acid-inducible gene I) encoded by the DDX58 gene in the human genome (1, 2) and MDA5 (Melanoma Differentiation-Associated protein 5) encoded by the IFIH1 gene (3, 4) are known as important protein initiators of earliest immune responses to viral infection. A relatively large body of work has focused on understanding their roles in triggering the same innate immune pathway as they indeed share many similarities at a structural and functional level. However, it is becoming increasingly clear that there are unique differences between RIG-I and MDA5, such as their activation mechanisms and contextual functionalities, that need to be considered in order to fully appreciate their individual function. A comprehensive analysis of multiple aspects of RIG-I and MDA5 from their evolutionary origins and behavior among different species to their structures and molecular signaling will allow for a more nuanced understanding of their functional purposes.

## Functional Similarities and Differences Between RIG-I and MDA5

**Figure 1:**
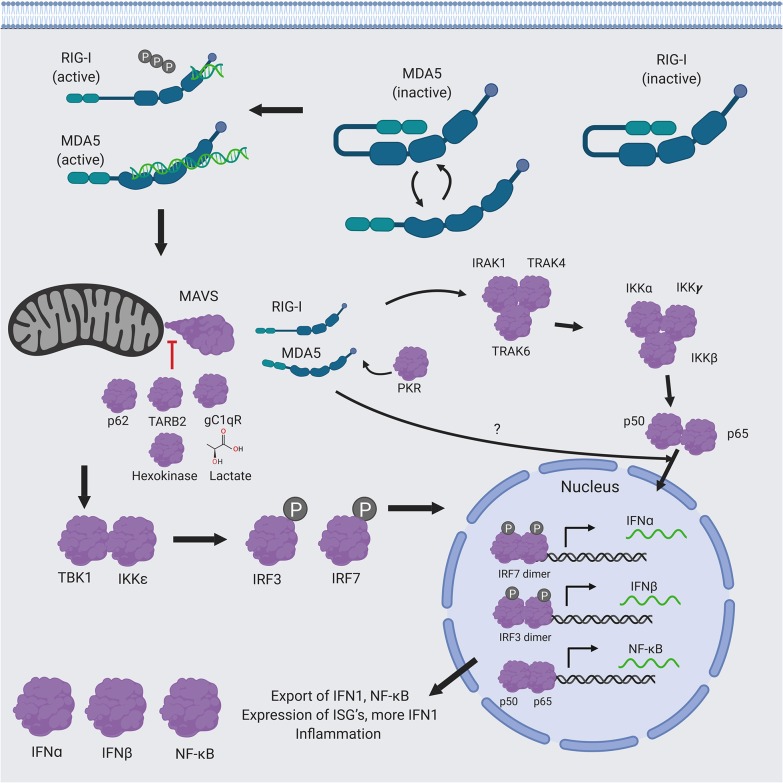
RIG-I/MDA5 signaling pathway RIG-I and MDA5 are first activated by recognition of PAMP dsRNA, which causes them to interact with MAVS. Following the activation of MAVS by RIG-I/MDA5, a molecular cascade involves the interaction of IKKε and TBK1, which is followed by phosphorylation of the transcription factors IRF3 and IRF7, ensure to translocate the phosphorylated p-IRF3 and p-IRF7 into the nucleus, where they dimerize and bind to transcription factor binding sites of the IFNα and IFNβ genes to activate their transcriptions. Expression and exportation of these genes into the cellular milieu trigger the IFN1 signaling cascade in an autocrine or paracrine fashion to induce expression of hundreds of interferon stimulated genes (ISGs) and inflammatory genes to confer antiviral resistance. RIG-I and MDA5 also activate the NF-κB pathway. RIG-I appears to act upstream of the canonical pathway, which results in the translocation of the two functional NF-κB units (p50 and p65) into the nucleus, while MDA5 appears to affect NF-κB expression independently from this pathway. Figure created using BioRender software.

The innate immune response is a combination of non-specific defense mechanisms by the host that are critical for early detection and inhibition of pathogen growth before the adaptive immune response has time to produce proper cell-mediated immunity, such as the development of antibodies and cytotoxic T-lymphocyte responses (CTL) against the invading pathogen and/or the pathogen-infected cells (5). Cells of the innate immune arm, such as leukocytes and epithelial cells, are able recognize general components of the microbes (e.g., viruses) that are shared among related microbes. These microbial structures are called pathogen-associated molecular patterns (PAMPs) (e.g., viral dsRNA) that are specifically recognized by the cellular pattern recognition receptors (PRRs) (e.g., RIG-I, MDA5, or Toll-like receptors TLRs) which are then activated (Figure 1). The specific signaling mechanisms of RIG-I and MDA5 activation will be discussed in detail below. Here, the cascade of event leading to IFN1 production is briefly summarized. Upon binding to PAMP (e.g., dsRNA), the activated RIG-I and MDA5 interact with the mitochondrial antiviral signaling proteins (MAVS), which forms a multilayered protein complex contain several different proteins (6–9). The MAVS complex then catalyzes the interaction of inhibitor of nuclear factor kappa-B kinase subunit epsilon (IKKε) and the serine/threonine-protein kinase 1 (TBK1) (10–12), which phosphorylate the transcription factors interferon regulatory factors 3 and 7 (IRF3 and IRF7) (13). Phosphorylated p-IRF7 (14) and -pIRF3 (15) factors then dimerize and translocate into the nucleus, where they activate the expression of the type 1 interferon genes (IFN1: IFNα and IFNβ). IFN1 proteins are then exported out of the cell to activate IFN1 signaling cascade by binding to their receptor (the IFNα/β receptor or IFNAR) either on the same cells or neighboring cells in an autocrine or paracrine fashion. This results in the production of more IFN1 (in a positive feedback loop) and a variety of interferon-stimulated genes (ISGs), which mediate vasodilation near the site of the pathogen infection and uptake of fluid, recruitment of innate immune cells, such as macrophages, neutrophils, and dendritic cells to the site of the infection that is aided by chemokine gradients to mediate innate immune cell-mediated killing of the infected cells (16).

**Figure 2:**
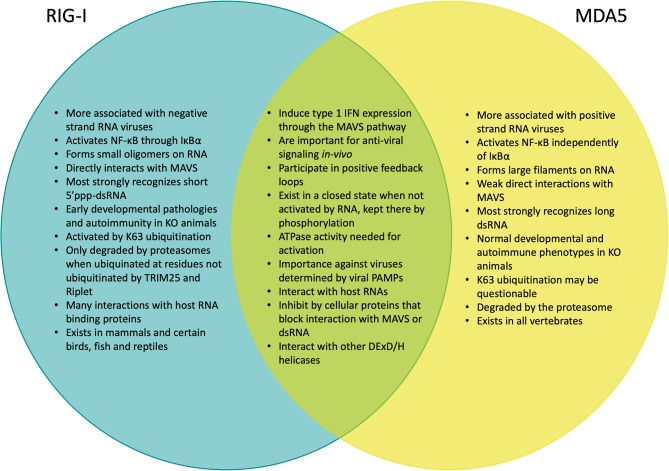
Venn diagram comparing the signaling and functional similarities and differences between RIG-I and MDA5.

RIG-I and MDA5 appear to differentially induce IFN1 in response to different viral pathogens (17), with RIG-I generally responding most potently to negative-strand RNA viruses, such as influenza viruses (18, 19), bunyaviruses (20, 21), filoviruses (22), and rhabdoviruses (18, 23) as well as the positive-stranded Japanese encephalitis virus (18), while MDA5 is activated during infection by positive-strand picornaviruses (18, 24, 25) and arteriviruses (26, 27) as well as by hepatitis D virus (28), Kaposi's sarcoma-associated herpesvirus (KSHV) (29). RIG-I and MDA5 may also play a role in recognizing non-viral pathogens, as MDA5 has been found to respond to malaria (30) (Figure 2). Neither are individually critical in reovirus (24) and in dengue virus infection (24, 31) but the presence of either in combination with Toll-like receptor 3 (TLR3) is critical to have effective anti-viral repsonses (32). Each serves an additive role during West Nile virus infection (33), which is likely mediated by the production of multiple PAMP species in the infected cells (34). Indeed, RIG-I and MDA5 have also been shown to recognize different sections of the same viral genome due to their differing preferences for RNA binding (35), illustrating how RIG-I and MDA5 can act both independently and synergistically. This has also been shown functionally in viruses where both RIG-I and MDA5 have been found to be essential to induce the necessary levels of IFNβ signaling for antiviral control against paramyxovirus (18, 36–38) and rotavirus infections (39).

While RIG-I and MDA5 participate in the IFN1 signaling pathway (40), it is clear from animal modeling that they might be functionally distinct. While C57BL/6 MDA5 KO mice exhibit no obvious phenotypes (18), C57BL/6 RIG-I KO have high embryonic lethality as they don't live past 3 weeks of birth and experience growth retardation and liver degeneration (18, 41). Furthermore, when RIG-I KO mice are back crossed onto the more genetically flexible 129S1 strain (18), these mice can spontaneously develop colitis symptoms (42). Clinical cases with mutations in RIG-I and MDA5 have distinct autoimmune presentations, with RIG-I mutations being associated with atypical Singleton-Merten Syndrome, while MDA5 mutations have been linked to classical Singleton-Merten Syndrome, Aicardi-Goutières syndrome, Systemic Lupus Erythematosus, Type 1 Diabetes and Graves disease (43, 44) (Figure 2). There is growing evidence that overt innate-immune interferon signaling plays a critical role in the development of other forms of autoimmune conditions (45). Taken together, this suggests that RIG-I and MDA5 may differ significantly in their roles during development as well as in responding to different types of viral infection that is partially dependent on the PAMPs that are available in any given context.

There is also increasing evidence that RIG-I and MDA5 have additional distinct molecular functionalities in immune signaling (43). It is well-established that the interferon regulatory factor (IRF) and innate immune NFκB cytokine signaling pathways have many areas of cross-regulation and expression (46). Accordingly, both RIG-I and MDA5 have been shown to activate NFκB signaling during RSV infection, but only RIG-I appears to act upstream of the canonical IκBα-NFκB pathway (47, 48) (Figure 1). While both are known to activate NFκB mediated expression of IL-6 and pro-IL-1β through the interaction of CARD9 with BCL10 (49, 50), the independence of MDA5 from the IκBα pathway suggests that it influences NFκB signaling in other as yet uncharacterized ways (43). A possible explanation for MDA5's independence from the IκBα pathway may be that MDA5-mediated NFκB (but not IRF) signaling requires TRIM25, which activates RIG-I by ubiquitination (to be discussed in detail below). This potentially implicates TRIM25 in other mechanisms besides activating RIG-I (51, 52). RIG-I (but not MDA5) also induces inflammasome assembly-mediated cleavage and maturation of pro- IL-1β by caspase 1 (24, 34, 53). Finally, RIG-I has been shown to inhibit RNAi complexes mediated by the endoribonuclease Dicer, which is encoded by the DICER1 gene and cleaves dsRNA and pre-micro RNA into short single-stranded RNA fragments known as small interfering RNA (siRNA) and microRNA (54), by interacting with the probable ATP-dependent RNA helicase DHX58 (also known as the Laboratory of Genetics and Physiology 2 LGP2 protein), which inhibits Dicer (55) as well as the Dicer-complex protein TRBP (56). LGP2 has been shown to exhibit conflicting effects on RIG-I and MDA5 signaling (57–59), and future studies are needed in order to clarify these regulatory mechanisms.

## Structures of RIG-I and MDA5

**Figure 3:**
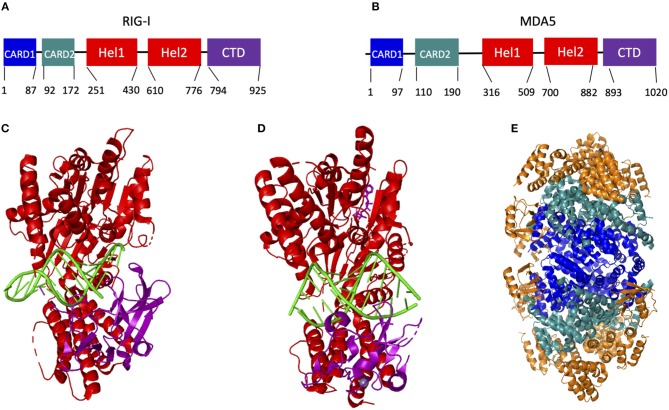
Organization and known structures of RIG-I and MDA5. **(A,B)** Graphic representing the domain organization of RIG-I **(A)** and MDA5 **(B)**. **(C–E)** Known structures of human RIG-I and MDA5, with X-ray crystallography structures of RIG-I Hel-CTD **(C)** and MDA5-Hel-CTD **(D)** interacting with dsRNA, and CARD1/2 of RIG-I interacting with the MAVS CARD domain **(E)**. In **(C–E)**, the helicase domains are shown in red, the CTD in purple, RNA in green, the CARD1 domain in blue, the CARD2 domain in aquamarine and the MAVS CARD domain in orange **(C)** was adapted from reference (61), **(D)** from reference (62), and **(E)** from reference (63).

RIG-I and MDA5 are expressed in all cell types (60), but are most well-known for their functions inside innate immune cells, such as macrophages, neutrophils, and dendritic cells, as well as in other cells like mucosal epithelial cells. They are classified as ATP-dependent DExD/H box RNA helicases. Their structure is highly helical and consists of two caspase activating and recruiting domains (CARD) at the N terminus of ~85 amino acids each, followed by a flexible hinge region and the helicase domain that consists of the RecA-like Hel1 and Hel2 domains with an ATP binding and hydrolyzing domain at their interface (Figures 3A,B). In particular, the structure of the ATP binding site distinguishes RIG-I and MDA5 from other helicase proteins, such as Dicer. Unlike other DExD/H box helicases where RNA binding catalyzes the ATP binding site to become structurally organized, the ATP binding site in RIG-I and MDA5 remains comparatively open and structurally dynamic following RNA binding. This is aided by the ATP binding site being formed by an interface between the two Hel domains, which are relatively far apart (64).

These structural features are connected by another flexible hinge region to the unique and predominantly β-sheet C terminal domain (CTD), which recognizes and binds to RNA (65). The CTD in RIG-I and MDA5 contains a zinc binding domain that is related to those of the GDP/GTP exchange factors (66). Each protein also contains a positively charged groove within this domain that recognizes dsRNA and this groove is structurally unique in each protein, potentially explaining their different RNA binding preferences (66). RIG-I primarily recognizes short double-stranded RNA with 5′ triphosphate groups (67–75), while MDA5 primarily recognizes long double-stranded RNA (76–79) (to be discussed in detail below.) It is notable in this regard that the Hel-CTD motifs adopt different orientations relative to dsRNA in RIG-I and MDA5. Specifically, the RIG-I Hel-CTD domain is tilted relative to dsRNA with the CTD interacting with the 5′ and 3′ ends of the dsRNA (61), whereas the MDA5 Hel-CTD domain runs parallel to the RNA strand (Figures 3C,D).

## Activation of RIG-I and MDA5 by Post-translational Modifications

**Figure 4:**
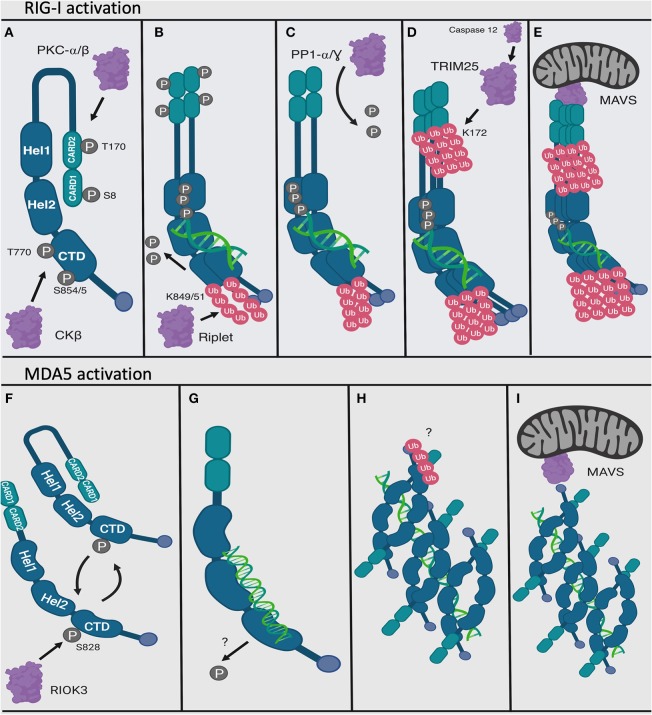
Activation mechanisms of RIG-I and MDA5. RIG-I and MDA5 are activated by interacting with viral dsRNA at the C terminal domain. In their endogenous and inactivated state, RIG-I and MDA5 are phosphorylated at their N and C terminal domains **(A,F)**. MDA5 may exist between open and close forms in its inactivated state **(F)**. Upon recognizing PAMP dsRNA, the C terminal domain becomes dephosphorylated and ubiquitinated for RIG-I and dephosphorylated for MDA5 **(B,G)**. RIG-I also dimerizes **(B)**. Next, RIG-I oligomerizes **(D)** and MDA5 forms longer filaments on dsRNA **(H)**, and the N terminal CARD domains of RIG-I becomes dephosphorylated **(C)** then ubiquitinated **(D)**. Finally, the CARD domain of the RIG-I oligomers interacts with the mitochondrial protein MAVS **(E)**, and the MDA5 dsRNA filaments also activate MDA5 (though it has a weaker CARD-CARD interaction with MAVS) **(I)**. Figure created using BioRender software.

The series of steps required for RIG-I and MDA5 activation have been described in depth elsewhere (80–84). Briefly summarized, these proteins endogenously exist in the cytoplasm of the cell in a phosphorylated and inactivated conformation when they are not activated by PAMP (dsRNA) (85–87) (Figures 4A,F). Phosphorylation is mediated at the N terminal CARD domains (S8 and T170) of RIG-I by PKC-α/β (88, 89) and at the C terminal RNA interaction domain (S854, S855, and T770) by CKβ (90). On the other hand, MDA5 is phosphorylated at S828 by RIOK3 (91) as well as by other yet unknown kinases (92, 93). RIG-I is also acetylated at K909 in its C terminal domain that requires deacetylation by HDAC6 to be able to recognize RNA in its activated form (94). Upon recognition of PAMP (dsRNA), RIG-I unfolds into an open and activated state that is mediated by the flexible hinge regions between the CARD domains and the helicase domain, and between the helicase and the C terminal domain (64, 87, 95–98) (Figure 4B). On the contrary, there is evidence to suggest that MDA5 has a more dynamic structure (99). Unlike a model of RIG-I activation described above, MDA5 exists in a conformational equilibrium between close and open forms, with close forms favored in the dsRNA unliganded state. While not yet formally demonstrated, it is possible that MDA5 may be inhibited in the absence of the dsRNA ligand by its structural dynamics, which may prevent strong protein-protein interactions (Figure 4F). However, upon binding to dsRNA ligand, MDA5 adopts an open and activated form, which is perhaps more conducive for protein-protein interactions (Figures 4G,H).

Once the C terminal domains have been de-phosphorylated, the E3 ubiquitin ligase Riplet attaches ubiquitin peptides onto the C terminal domain of RIG-I at residues K849 and K851 (100, 101). It was previously shown that ubiquitination by Riplet was necessary for opening RIG-I and for ubiquitination of the CARD domain (102). However, *in-situ* studies found that dsRNA was sufficient to weaken the interaction between purified RIG-I C terminal domain and RIG-I CARD domains (86) and that dsRNA was necessary for Riplet ubiquitination (103), calling into question the sequential order for RIG-I activation (Figure 4C). Following de-phosphorylation of the CARD domain by the phosphatase PP1-α/γ (92), this domain is polyubiquinated at K172 by the E3 TRIM25 ubiquitin ligase (104), which itself is activated by Caspase 12 (105) (Figure 4D). TRIM25 interacting with RIG-I may also be mediated by their mutual interactions with certain host long non-coding RNA (lncRNA), which occurs outside of the dsRNA recognizing domain in the CTD of RIG-I (106).

A recent study showed that Riplet rather than TRIM25 was primarily responsible for ubiquitinating and activating RIG-I (103). However, there are several factors to take into consideration with this study. These recent results were obtained using KO 293T and mouse embryonic fibroblast (MEF) cells and that it was not clear whether K63 ubiquitination occurred at other known lysine sites in RIG-I. The question remains whether Riplet can ubiquitinate other lysine residues in the absence of TRIM25. Additionally, *in-situ* experiments comparing RIG-I ubiquitination by Riplet and TRIM25 utilized an E2 enzyme (103) that had been found to be specific for Riplet (107). While the E2 that utilizes TRIM25 has not yet been identified, TRIM25 has been shown to ubiquitinate RIG-I *in-situ* when a general mixture of E2 proteins was used (108). The protein levels of TRIM25 may also have to be at a certain level in order for it to productively ubiquitinate RIG-I, as the ubiquitin protease USP15 deubiquitinates TRIM25 at later time points in viral infection (109).

Finally, TRIM25 has been found to be essential for RIG-I activation and IFN signaling *in-vitro* and *in-vivo*. For the former, siRNA-mediated knock-down (110, 111), cellular knock-out (112) and inhibition by viral protein (109, 113–116) conditions for TRIM25 in multiple cell types have been shown to change RIG-I cellular localization (110) and to negatively affect RIG-I K63 ubiquitination, association with MAVS and IFN signaling [when the constitutively active RIG-I CARD domain was overexpressed (109, 112–116) or during viral infection (109, 111, 114)]. Viral inhibition of TRIM25 may even be a source of a positive selection during the evolution of certain viruses, as NS1 IAV proteins have been found to interact with species specific TRIM25 (114). For the latter, MEFs from TRIM25 KO mice have significantly downregulated IFN1 production upon viral infection (113) and KO mice for NLRP12, which is a competitive interactor with TRIM25 to RIG-I, show increased interferon production and more resistance to viral infection (117). The known contributions of TRIM25 to innate immunity have recently been summarized elsewhere (52).

It is clear that both Riplet and TRIM25 can mediate K63-linked polyubiquitination. However, it has also been found that *in-situ* incubation of purified RIG-I CARD domains with ubiquitin can be activated by free and unlinked K63 polyubiquitin chains (118), calling into question whether TRIM25 only attaches K63-linked ubiquitin motifs to RIG-I-CARD or if it also catalyzes the formation of unlinked K63 polyubiquitination chains (119). A possible explanation for these differing results is that RIG-I has been shown to be covalently K63 ubiquitinated by TRIM25 when analyzed by mass spectrometry from cells (104), while experiments that demonstrate non-covalent K63 ubiquitination are those involve primarily interactions with purified proteins.

It has also been recently found that RIG-I is K63 ubiquitinated at K164 and that it may be functionally redundant to K172 (120, 121), with their ubiquitination possibly upregulating the K63 ubiquitination of the other 6 lysine residues in RIG-I (121). However, it is unknown whether TRIM25 ubiquitinates K164 or any of the other RIG-I lysine residues. Notably, these additional lysine residues in the CARD and C terminal domains of RIG-I and MDA5 are known to be K27 and K48 ubiquitinated [which are associated with degradation of RIG-I (122, 123) and MDA5 (123)], but the four listed above appear to be the essential residues for activation of RIG-I (122, 124).

The presence of K63 ubiquitin modifications on MDA5 is more controversial. Independent studies have found that MDA5 is (125, 126) or is not (126) K63 polyubiquitinated. It has also been independently found that TRIM25 does not affect ubiquitination of MDA5 (without distinguishing between K63 and K48 polyubiquitination) (104) and for TRIM25 to increase K63 ubiquitination (125), the only apparent difference in the experimental models being the usage of HEK293T (104, 126) vs. HEK293 (125) cells. TRIM65 has also been recently found to be essential for MDA5 activation by K63 polyubiquitination at K743 (127). It is clear that additional studies are needed in order to clarify the ubiquitination mechanisms of MDA5.

## Oligomerization and Filamentation to Activate RIG-I and MDA5 Functions

Upon binding to PAMP (dsRNA), RIG-I oligomerizes with other RIG-I/dsRNA complexes to form helical oligomers (128) in a 2:2 complex using the purified RIG-I protein (87), where the activating ubiquitin motifs serve as a scaffold to link the oligomers together (118). These oligomers have been found to be necessary under normal conditions to activate RIG-I. This may be due to the helical structure of the RIG-I oligomers closely matching those formed by MAVS (63), which is known to form filaments *in-vitro* (129, 130) mediated by its own CARD domains (131, 132). A structural model of MAVS activation by RIG-I has been proposed of stacking MAVS CARD domains on top of RIG-I CARD domains to extend the RIG-I helix (133).

The minimum length of dsRNA found to activate RIG-I is 13 base pairs, which is equivalent to the minimum length to facilitate the formation of a 2-RIG-I/dsRNA dimer (75). That being said, shorter (~10 bp) 5′ppp stem loop dsRNA complexes that have previously been used to obtain X-ray crystallographic structures of RIG-I interacting with dsRNA (61, 134, 135) (Figure 3C) can also activate IFNβ signaling in cells (135, 136) and in mice (136). Furthermore, A549 cells that were transfected with RIG-I plasmid 6 h prior to RNA transfection had a minimum dsRNA length of only 8–10 bp required for activation (75). This indicates that RIG-I oligomerization may not be necessary for activation of the IFNβ pathway under some experimental conditions, which need to be further investigated.

MDA5 has also been shown to oligomerize to form long RNA-associated filaments *in vitro* (62, 137, 138) (Figures 3D, 4H), which may be aided by chaperone proteins (139). Given that the K743 residue found to have been ubiquitinated by TRIM65 (127) is located on the surface of Hel2, it is possible that K63 ubiquitin residues may also help stabilize MDA5-dsRNA filaments (140). However, MDA5 also spontaneously forms filaments and induce MAVS to form filaments independently of ubiquitin *in-situ*. It is also thought that the formation of longer filaments by MDA5 may be mediated by a longer linkage region between CARD2 and Hel1 than in RIG-I by 50 amino acids (the length of which is well-conserved across species), allowing for the association of more CARD domains in an oligomer (133).

The formation of longer filaments by RIG-I has been more controversial, giving rise to two alternate models of RIG-I activation: formation of individual single unit of RIG-I with short dsRNA monomers (leaving a free dsRNA end, such as a hairpin loop), which then oligomerizes via CARD tetramerization that is linked by their ubiquitin chains, or filamentation on longer dsRNA. Like MDA5, RIG-I can form filaments *in-situ* independent of ubiquitin (141, 142) and induces MAVS to also form filaments (142), and MAVS is known to form filaments *in-vitro* (129, 130) mediated by its own CARD domains (131, 132). However, RIG-I filamentation on an RNA template (forming “beads on a string”) as opposed to smaller-scale oligomerization hasn't yet been shown to occur *in-vitro*. Part of the reasons for the suggestion that RIG-I was strongly activated by shorter dsRNA was based the comparison on mass equivalents of RNA species as there were less 5′ triphosphorylated ends for longer dsRNAs with greater mass than shorter dsRNAs with more 5′ triphosphorylated ends (76). However, when RNA species were normalized by molar equivalence, dsRNA length appeared to be positively correlated with RIG-I signaling (141–143), which became insignificant at around 500 bp (141, 143). It is significantly shorter than the length of dsRNA that activates MDA5, which forms filaments on 2,000 bp dsRNA (137). The kinetics of RIG-I and MDA5 interacting with dsRNA (which will be discussed in detail below) might possibly explain the decrease in dsRNA length efficiency to activate RIG-I as compared to MDA5, as RIG-I seems to first recognize the 5′ppp end before sliding down the length of the dsRNA (144), whereas MDA5 dynamically associates and disassociates along the length of long dsRNA (137). Meanwhile, it is still unclear whether RIG-I can preferentially be activated by longer dsRNA independently of its unknown ability to form filaments *in-vitro* (145).

## Modes of RLR-MAVS Interaction and RLR Downstream Signaling

Once fully activated and oligomerized, the RIG-I CARD domain can then interact with MAVS (146–149) (Figures 4E,I), which is part of a protein complex containing a variety of other cellular proteins (6–9). While the MDA5 CARD domain has much weaker direct association with MAVS than the RIG-I CARD domain, it is sufficient to lead to its activation and potentiates activation of MAVS by RIG-I (146), the mechanisms of which have yet to be determined. The activated MAVS complex then initiates a molecular cascade which eventually results in expression of IFN1 (150) (Figure 2).

Interestingly, full length RIG-I, when overexpressed, has been found to associate with MAVS in the absence of activating dsRNA and the interaction can be ablated by phosphorylation at S8 and T170 (87), suggesting that the CARD phosphorylation sites function at least in part to prevent association of the inactive form of RIG-I with MAVS. Furthermore, the crystal structure of the interaction between the RIG-I CARD and MAVS CARD domains shows the RIG-I CARD2 domain (92–173) interacting with MAVS CARD domains on the outside of the tetramer and the RIG-I CARD1 (1–87) domain facing toward the center of the tetramer (63) (Figure 3E). NMR solution structures of RIG-I CARD2 also shows that T170 (which is required for dephosphorylation by PP1-α/γ) is largely buried within the CARD2 domain in a section that would be in closer contact with the helicase domains, suggesting that dephosphorylation of T170 affects an interaction domain between CARD2 and the C terminus (151). Furthermore, NMR of a C terminal construct of RIG-I with the CARD2 domain shows stable interactions of CARD2 and the C terminal domain (151). What all this may mean is that, while the CARD1 domain of RIG-I is somewhat exposed in its inactivated form and therefore can be shown to interact with MAVS, full exposure and engagement of both RIG-I CARD domains (CARD1 and CARD2) with the CARD domain of MAVS is necessary in order to induce IFN1 signaling. The CARD domains of RIG-I also appear to be generally structurally stable, as electron microscopic structures have been obtained of the full length RIG-I bound to blunt-ended dsRNA showing both CARD domains exposed (87). On the contrary, the CARD domains of MDA5 may be comparatively more flexible than those of RIG-I in order to mediate long MDA5-dsRNA filament formation (99).

The activated MAVS complex induces association of the inhibitor of nuclear factor kappa-B kinase subunit epsilon (IKKε) and the serine/threonine-protein kinase 1 (TBK1) (10–12), which collectively phosphorylate the interferon regulatory factors 3 and 7 (IRF3 and IRF7) (13) (Figure 1). IKKε and TBK1 also interact with a number of other co-factors (152, 153), such as the DEAD-box helicase 3 (DDX3) (154). The activated p-IRF3 (15) and p-IRF7 (14) then translocate into the nucleus and dimerize, where they then act as the primary transcription factors for IFNα and IFNβ, respectively. Existing evidence suggests that IFNα is more primarily produced in the earliest time points following RIG-I/MDA5 activation, while IFNβ is produced later and is responsible for more robust anti-viral control throughout the innate immune response period (155). There is also a distinction between innate immune cell types for IFN1 production, as cells like fibroblasts and conventional dendritic cells produce IFNα and IFNβ (41, 156), while neutrophils only produce IFNβ (157) and plasmacytoid dendritic cells only produce IFNα primarily through the TLR signaling pathways (41, 158). Signaling through RIG-I is also known to be essential for the process of TLR-mediated phagocytosis by macrophages (159).

Interferons are then secreted out of the cell, where they bind to their own receptor (IFNAR) and activate the Janus kinase/Signal Transducer and Activator of Transcription proteins (JAK/STAT) signaling pathways, which result in a positive feedback signaling loop to further increase RIG-I/MDA5 expression and activation (160) and IFN1 production (161, 162). Expression levels of RIG-I and MDA5 have consistently been found to be upregulated downstream of type I (163, 164) and type II (165, 166) IFN signals. MDA5 upregulation has additionally been found to occur independently of cytokine expression at least during picornavirus infection (167).

## Specific RNA Features Recognized by RIG-I and MDA5

**Figure 5:**
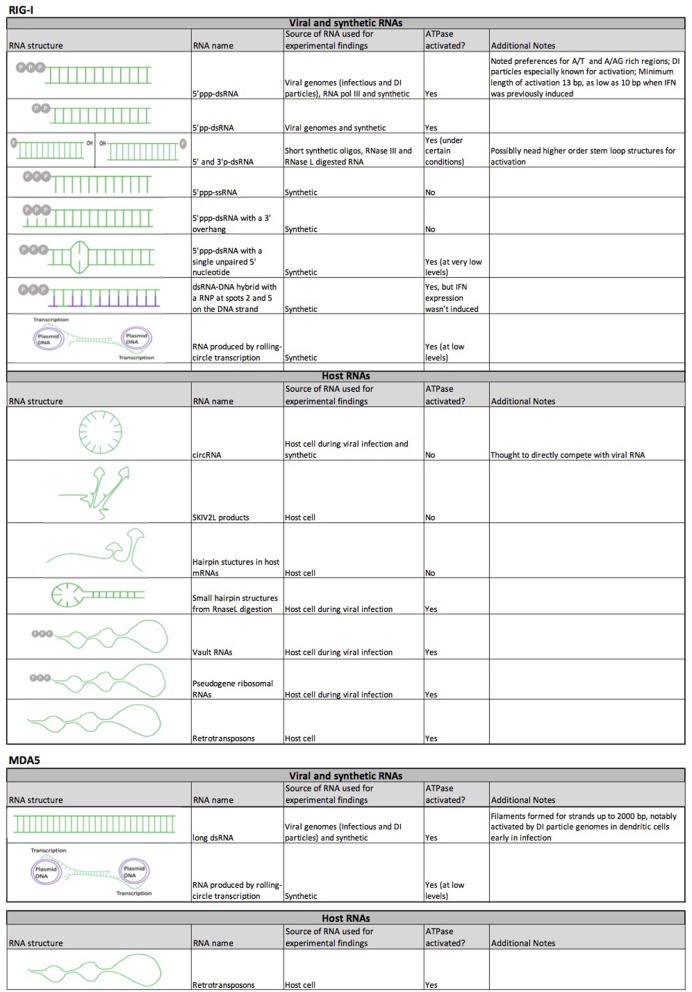
RNA species that interact with RIG-I and MDA5. Table summarizes the general structural features of RNA species, their source during experimental studies and their ability to activate the ATPase functions of RIG-I and MDA5. RNA constructs are shown in green, and DNA constructs in purple.

One of the most obvious distinctions between RIG-I and MDA5 is in the RNA species to which they bind for activation (Figures 1, 5). RIG-I has the highest affinity for short dsRNA that is tri-phosphorylated at the 5′ end (67–75), with RIG-I having been found to directly interact with the 5′ tri-phosphate group of the dsRNA (71, 73). While RIG-I can bind to ss-5′ tri-phosphorylated RNA (69), RIG-I cannot be activated by it (69, 168, 169), likely due to a conformational need to recognize double-stranded RNA. As a result, RIG-I is greatly attenuated by a 5′ overhang as well as those with a 3′ overhanging the 5′ tri-phosphate end (170). In fact, a single unpaired 5′ tri-phosphorylated nucleotide is sufficient to competitively inhibit RIG-I, which has been exploited by RNA viruses to evade RIG-I recognition and IFN1 signaling (171). The unique preference of RIG-I for 5′ tri-phosphorylated RNA can be explained by the specific orientation that the RIG-I C terminus adopts when directly interacting with the 5′ tri-phosphate group of the 5′ tri-phosphorylated dsRNA (71, 73) as compared to unphosphorylated blunt-ended dsRNA (172).

The minimally required and exclusionary features of the 5′ and 3′ dsRNA ends for RIG-I activation have proven to be complex. Certain studies suggest that a 5′ diphosphate group is the minimum feature required for RIG-I binding and activation, with 5′ monophosphate dsRNA failing to productively activate RIG-I as compared to 5′ di and tri-phosphate dsRNA (173). Additionally, RIG-I poorly distinguishes between dsRNAs with either 5′ tri-phosphate and 5′ diphosphate group. When the free energies of each interaction are calculated, the affinity for 5′ triphosphate being lowered by disassociation of magnesium from the RIG-I/dsRNA complex. Both are significantly more favorable for binding RIG-I monophosphate dsRNA (174). This similarity in affinity appears to be important in the context of infection with viruses that produce 5′ diphosphate RNAs, such as reoviruses (173). Likewise, the difference of energic binding between monophosphate dsRNA and bi- and tri-phosphate dsRNAs is likely important for distinction between self (host) and non-self (foreign) RNA, the mechanisms of which will be discussed in detail below. The ATP hydrolysis functions of RIG-I have been shown to drive rapid disassociation from certain RNA features, such as 5′ monophosphate dsRNA (174, 175) and 5′OH RNA (144, 176), which is particularly important for 5′ monophosphate dsRNA because it is found in mRNA after decapping during the mRNA degradation process (177).

On the other hand, other studies have shown that RIG-I can interact with monophosphate dsRNA to a certain degree, as has been found to be the case for short synthetic dsRNA with a 5′ and 3′ monophosphate group (69), poly(I:C) digested with RNase III (76) [which generates 5′ mono-phosphate/3′-OH dsRNA (178)] and HCV RNA (179) and mitochondrial RNA [in the p53 deficient mice (180)] digested with RNase L [which produces 5′ OH and 3′ mono-phosphate dsRNA at subnanomolar levels (181), as has been found to be the case for HCV RNA (179).] It appears that the 5′ monophosphate is the determinate feature for RIG-I activation independently of the 5′ or 3′ OH group in all these cases. A possible explanation for the discrepancy between the studies was that higher order RNA structures might compensate for the less optimal 5′ and 3′ ends, as monophosphate dsRNA that did not contain stem-loop structures did not activate RIG-I and RNA regions repetitive in certain nucleotides had been found to be critical for RIG-I activation (179). Future studies are required to further characterize the behavior of RIG-I with these RNA species.

As previously mentioned, MDA5 preferentially associates with long dsRNA (76–79). The crystal structure and molecular modeling of MDA5/dsRNA complex suggest that it can recognize the entire first turn of the blunt-ended dsRNA (182) in a similar way as LGP2 can (183). Like RIG-I and MDA5, LGP2 belongs to the ATP-dependent DExD/H box RNA helicases (184), which is structurally similar to RIG-I and MDA5 but lacks the CARD domains at the N terminus (185). MDA5 has also been found to be activated by the digested products of RNase L specifically from parainfluenza virus (186).

The presence of certain repetitive RNA elements appears to be another contributing factor in determining interaction of RNA with RIG-I and MDA5, which has recently been described in detail elsewhere (187). While RIG-I and MDA5 are mostly implicated in the immune response to RNA viruses, it has also been found to be activated by 5′ tri-phosphorylated dsRNA intermediates generated by cellular RNA polymerase III from AT-rich DNA sequences (188) and during infection with Epstein-Barr virus (a DNA virus) (189). RIG-I has additional binding preferences for certain nucleotide motifs, such as uridine-rich 5′ tri-phosphorylated hairpin RNA (190), synthetic AU- rich hairpins (191) and those naturally found in the genomes of Sendai virus defective-interfering (DI) particles (192), measles (193), Influenza A virus (IAV) (194) and in KSHV RNA transcripts (195), and poly (U/UC) regions (196) and poly (A/AG) regions (197) in the antisense Hepatitis C virus (HCV) genome. It is of particular interest that the poly (A/AG) HCV regions are located significantly downstream of the 5′triphosphate group (197), thus potentially implicating other parts of RIG-I (e.g., helicase domain) as potential RNA interacting domains. Repetitive RNA elements may also be important in allowing for interaction of inhibitory RNAs that do not have 5′ or 3′ features needed for full activation of RIG-I, as has been shown to be the case with GA-rich regions in circular long-non-coding RNA lnc-Lsm3b (198). These specific interactions explain their primary role as anti-viral receptors, as these viral motifs are mostly not found in cellular RNAs (199).

RIG-I and MDA5 have been particularly implicated in their response to RNA genomes of viral defective interfering (DI) particles, as these defective viral genomes (DVGs) have originally been found to induce interferon signaling (150). DI particles are produced by many viruses during infection, and while they are similar in many regards to standard viral particles, such as in appearance and composition, they cannot productively infect cells (200). This is largely thought to be due to the presence of large and deleterious deletions in the DVG of DI particles (201). Some DVG RNAs have also been noted to have “copy-back” motifs in which one end of the genome can base pair with an inverted copy at the opposite end of the genome, which may be due to stalled and aberrant replication (202, 203).

Copy-back RNA motifs specifically seem to be important for RLR activation in that they tend to contain hairpin motifs and 5′ tri-phosphate groups, as has been found for Sendai (204–206), measles (35, 207), and chikungunya (35) DVG RNAs in activating RIG-I. In the case of IAV, DVG RNAs might even be more potent activators of RIG-I than the full-length viral genome. Cells that were blocked from viral protein synthesis experienced RIG-I mediated IFN1 expression when infected with IAV stocks grown in chicken embryonic eggs (which produced higher relative quantities of DI particles with DVG RNAs) but not with IAV grown in cell culture, indicating that RIG-I activation by the genomes from primarily non-DI IAV particles may require active viral RNA synthesis (208). A potential explanation to this observation is that RIG-I appears to be activated by the full viral genome via its panhandle structure, the affinity of which is lowered by the presence of mismatched and unpaired nucleotides in this region of the viral genome that is conserved across influenza virus strains (209). However, the overall panhandle structure is conserved between DVGs (205) and the full length viral genome (209), and deletions within DVGs are monogenic and internal (210). The specific molecular mechanisms of enhanced RIG-I signaling by IAV DVGs have yet to be elucidated, although the level of exposure of the panhandle may play a role. While the full extent of MDA5 interacting with DI RNA is currently unknown, MDA5 appears to be more predominantly activated by DVG RNA than RIG-I specifically in dendritic cells early in the viral infection cycle (211), which may be a contributor toward the phenomenon of DI particles enhancing dendritic cell maturation (212).

The comparative abilities for DI particles vs. infectious virions to activate RIG-I and MDA5 have important implications for understanding viral pathogenesis and for vaccine development. There is a burgeoning interest in this regard, especially in populations which are typically more challenging to achieve successful preventative vaccination, such as elderly populations with IAV vaccination (213). Elderly populations in general do not develop as strong of memory immune responses to vaccines as their younger counterparts (214–217) and have been found to have decreased RIG-I mediated IFN1 signaling (218). Correspondingly, the influenza vaccine has been shown to decrease in effectiveness in older populations as the influenza season progresses (219). A DI-vaccine that strongly activates innate immune cells and increases the adaptive immune response could therefore potentially boost the immune responses to vaccines in more vulnerable populations. Additionally, DI particles have shown to be an important contributor of viral persistence (200, 220, 221). This raises the question of whether a viral infection may alternate between producing primarily infectious virions which eventually activates the innate immune response and producing primarily DI particles which requires less cellular activity but may initiate an even stronger innate immune response (222–224). Taken altogether, DI particles provide yet another layer of distinction between RIG-I and MDA5 in terms of how each recognizes different species of dsRNA.

## Distinction of Self (Host) and Non-self (Foreign) RNAs by RIG-I and MDA5

The preference for specific RNA species by RIG-I and MDA5 allow for them to distinguish between viral RNA and host RNA in most circumstances (225), although the specific mechanisms of distinction are not as clear for MDA5 as for RIG-I. Studies from clinical cases of MDA5 mutations provide contradictory models, with certain mutations found in Aicardi-Goutières syndrome (AGS) increasing MDA5 avidity for self RNA (226) with Alu retroelements found to be significantly enriched for interaction with AGS MDA5 mutations (227). The modification of dsRNA by host cells may be a primary inhibitor of MDA5 activation by host RNA as knockout of adenosine deaminase (ADAR1), which weakens dsRNA structures, allows wild-type MDA5 to be activated by Alu retroelements (227). However, other MDA5 mutations decrease affinity for known MDA5 ligands and ATPase activity, yet still demonstrate increased IFNβ expression (228, 229).

For RIG-I, a highly conserved residue in the C-terminal RNA binding pocket (H830) has been found to sterically exclude canonical self-RNA by the means of the N_1_-2′O-methyl self-RNA motif, also known as Cap1 RNA (61, 230). This results in a low binding affinity of RIG-I to cellular Cap1 RNA and decreased ATPase activity as compared to PAMP (dsRNA) (61, 231). Flaviviruses take advantage of this precise discrimination by encoding a viral 2′-O-methyltransferase capable of N_1_-2′O-methylating its positive-strand RNA genome in order to evade RIG-I recognition and IFN1 activation (230). Conversely, the mutations E373A and C268F found in the RIG-I protein in patients with auto-immune disorder Singleton-Merten syndrome confer the ability of the protein to recognize Cap1 RNA and become activated by ATP dependent and independent mechanisms, respectively (232). Furthermore, the E373Q mutation of RIG-I, which was designed to constitutively bind ATP, was found to increase the affinity of RIG-I with ribosomal RNA (233). It is noteworthy that host RNA contains additional internal RNA modifications and non-Watson-Crick base pairing which can inhibit activation of the other known dsRNA-sensing protein, the interferon-induced double-stranded RNA-activated protein kinase (PKR) (234), and it is known that synthetic 5′ triphosphorylated RNA containing pseudouridine, 2-thiouridine or 2′-O-methylated uridine has significantly decreased ability to activate RIG-I (67), which has been demonstrated to occur by preventing RIG-I filament formation *in-situ* (142). N-6-methyladenosine (m6A) nucleotides, which are well-known nucleotide modifications among viruses (235), have also been found to ablate dsRNA binding to RIG-I (236).

It has been demonstrated that certain RNA-DNA hybrid constructs with ribonucleotides at positions 2 and 5 of the DNA strand can bind to RIG-I and activate its ATPase activity (75). ATPase activity is necessary for full activation of RIG-I and expression of IFNβ (75, 237), so the minimum requirement of a motif not found in host RNA for ATPase activity has significant implications for the distinction between self and non-self RNAs. Expanding on this observation, exogenous ATPase activity may also be sufficient to potentiate RIG-I and MDA5, as LGP2 ATPase mutant mice are significantly more susceptible to viral infection even in the presence of functional RIG-I and MDA5 (238). However, this model is further complicated by certain RNA-DNA hybrids that are able to bind RIG-I and activate ATPase activity, but don't induce IFNβ expression (75). It is currently undetermined whether such hybrids can sterically inhibit RIG-I due to the presence of mostly dNTPs or whether they inhibit RIG-I in a yet undescribed way.

Recent kinetic studies of RIG-I and MDA5 activation by PAMP (dsRNA) help illustrate how ATPase activity is critical for their function and distinction between host (self) and foreign (non-self) RNA. RIG-I binding to ATP is sufficient for interaction with dsRNA (144, 176). RIG-I ATPase activity is inhibited in the absence of PAMP (dsRNA) by a helical arm that blocks the ATPase site (239). Upon interaction with PAMP (dsRNA), the helical arm shifts and the two helicase domains are brought together to form an active ATPase site (239). RIG-I then catalyzes ATP to break the 5′ppp dsRNA interactions within seconds. ATP is then rapidly hydrolyzed to facilitate translocation of RIG-I to the opposite dsRNA end, after which the RIG-I oligomers can form (144). On the other hand, ATP hydrolysis drives rapid disassociation of RIG-I from host RNA features. These features include dsRNA with a 5′ monophosphate group (174, 175) that is found in mRNA after decapping during the mRNA degradation process (177) and 3′ overhang RNA (144, 170) found in miRNA (240) as well as other RNA motifs, such as 5′OH RNA (144, 176) found in bacteria (241). Furthermore, an impaired ATPase functionality increases the promiscuity of RIG-I binding these host RNA motifs (144, 176, 242).

Similar ATPase functions have been found during MDA5 filamentous formation. The C terminus of MDA5 is critical to form organized helical filaments (138) and ATP binding drives association and hydrolysis and disassociation from foreign dsRNA [with little coordination being observed between neighboring MDA5 proteins (137)] in a manner that involves MDA5 twisting along its flexible and hydrophobic interface domains (243). Taken together, ATPase activity may be directed toward rapid disassociation from host dsRNA and degradation of RNA-DNA hybrids, but primarily act on the translocation pathway upon interaction with PAMP (dsRNA). It is also possible that host and hybrid dsRNAs could inactivate RIG-I independently of their ability to bind the C-terminus and activate ATPase activity. This has been shown, for example, for a hybrid RNA that has one strand consists mostly of DNA except at positions 2 and 5, which appears to bind RIG-I and activate its ATPase activity but doesn't activate IFN1 signaling (75). Future studies are needed in order to determine these differential interaction mechanisms.

## Novel Mechanisms of Inhibition or Activation of RIG-I and MDA5 by Cellular RNAs

Contrary to the traditional paradigm, there is increasing evidence to suggest that RIG-I and MDA5 interact with certain host RNA motifs, resulting in auto-activation or auto-inhibition of the IRF pathway (Figure 5). One of the most strongly supported models is activation by host and viral circular RNAs (circRNA). Originally found in a variety of pathogen genomes, circRNAs in eukaryotic cells were first thought to be byproducts of the pre-mRNA splicing process. However, they have later been found to be produced by a non-canonical “backsplicing” process and there is increasing evidence to suggest that they play some important regulatory roles (244), suggesting that they may have specifically evolved for this purpose. RIG-I was first found to interact with circRNA produced *in situ* (245). Interestingly, the minimum component required for RIG-I activation is an intron of pathogenic origin to be spliced out during the circularization process. As human introns have been found to be associated with many RNA binding proteins, it is speculated that these proteins may have prevented circularization of this particular synthetic circRNA used in this study (245) and that host RNA binding proteins normally prevent endogenous circRNAs from being detected by the innate immune system. Nevertheless, some viral infections can potentially expose these endogenous circRNAs for immune detection, as has recently been found to be the case for a novel host-derived circRNA (lnc-Lsm3b) that is IFN-inducible and shows a down-regulation of its binding to host proteins during viral infection and therefore appears to compete with viral dsRNA as an inhibitor of the RIG-I signaling feedback loop (198). Similar inhibitory mechanisms have also been noted for RNA products of the exonuclease SKIV2L (246). Finally, recent studies have found that hepatitis C virus (HCV) infection increases the expression of certain cellular RNAs that can inhibit RIG-I function. HCV infection increased the mRNA levels of hepatic selenoprotein, which was able to bind to RIG-I through a hairpin structure and inactivated it during viral infection (247). Infection by HCV, vesicular stomatitis virus (VSV), or Sendai virus, or direct exposure of cells to type 1 and 3 interferons increases expression of the cellular long non-coding RNA (lncRNA), namely lncATV, which similarly inhibits RIG-I function by directly interacting with it in order to promote virus replication (248). In addition to the greatly increased implications of RIG-I and MDA5 modulation, these findings also have significant implications in characterizing new biomarkers of disease, as increased serum selenoprotein level has been found to significantly associate with treatment failure of anti-viral drugs in HCV patients, and can possibly explain the increased prevalence of type 2 diabetes in HCV patients (247).

Cellular RNA has also been found to activate RLR signaling during viral infection. Vault RNAs, which are transcribed from four genes and are normally found in large ribonucleoprotein complexes in cytoplasmic “vaults,” are significantly enriched for binding to RIG-I during infection with KSHV (29). This may be due partly to viral infection-induced reduction in the level of cellular triphosphatase DUSP11, which dephosphorylates the 5′ppp group on the vault RNAs, as they could only be immunogenic (in the absence of viral infection) by the addition of the 5′ppp group. RIG-I and MDA5 have also been found to be activated by RNA microparticles produced *in situ* by rolling circle transcription, generating tandem repeat RNA strands (249). Retrotransposons may also be able to activate both RIG-I and MDA5, as both can be activated by LINE1 RNA independently of DNA sensing mechanisms and retrotransposition (250).

Viral infections can also induce recognition of host RNAs. Herpes Simplex Virus 1 (HSV1) infection, for example, has been shown to induce translocation of the host pseudogene *RNA5SP141* ribosomal RNA into the cytosol to bind to RIG-I. Knockdown of *RNA5SP141* decreased cytokine signaling during infection with HSV and EBV as well as influenza A virus (IAV) (251). RIG-I has also been found to be activated by hairpin RNA structures generated by cleavage of RNA by RNase L, which has been demonstrated to occur during HCV infection (179) as well as from mitochondrial dsRNA produced in p53 deficient mice (180). The mitochondria, in particular, may be an important source of immunostimulatory host dsRNA. Viral infections are well-known to cause mitochondrial damage (252). Knockdown and hepatocyte-specific conditional KO of mitochondrial RNA degrading enzymes resulted in the increase of cytoplasmic mitochondrial dsRNA which was able to activate MDA5 (253). Additionally, extracellular vesicles (EV) secreted by apoptotic endothelial cells were found to contain long interspersed nuclear element (LINE) and short interspersed nuclear element (SINE) RNAs that are products of RNA polymerase III and were able to activate RIG-I signaling (254). Collectively, these findings demonstrate the many unique ways by which cellular RNAs can modulate RIG-I and MDA5 functions as well as the potential implications of RIG-I activation by pharmaceuticals as an anti-viral or generalized immunotherapy, though much caution and studies would still be needed to determine the appropriate levels of RIG-I and MDA5 activation.

## Viral Modulations of RIG-I and MDA5 Functions

Given that RIG-I and MDA5 are critical for activating expression of IFN1 during viral infection, there is much interest in studying the interactions of these cellular proteins with viral factors (RNAs or proteins), as the ability to modulate interferon expression is a major evolutionary driving force in viral evolution (255, 256). There are many mechanisms viruses have evolved to evade RIG-I and MDA5 signaling, which have been discussed at length elsewhere (257, 258). Such mechanisms are of particular importance to segmented RNA viruses, providing potentially more dsRNAs for RIG-I and MDA5 activation (259). IAV and the other orthomyxoviruses are unique in that they replicate in the nucleus of the cells (260), preventing the viral RNA from being detected by the PRRs. However, recent preliminary evidence seems to suggest that RIG-I may also endogenously be present in the nucleus and performs similar viral RNA binding and activation of the IFN1 pathway (261), yet this finding has yet to be replicated by other laboratories.

There is also increasing evidence to suggest that RNA processing is another mechanism of immune modulation. Certain bunyaviruses can cleave the 5′ tri-phosphate group from their genomic RNA (262) in order to avoid immune detection. RIG-I has also been found to be subjected to negative modulation by RNAi during IAV infection (263). On the contrary, nucleoproteins from the Sendai virus (264) regulate the number of DI particles being produced, and IAV nucleoproteins also regulate the production of abortive replication RNA (208), mini viral RNAs (265) and DVG RNA (208), all of which are immunostimulatory. The Semliki Forest virus (SFV) polymerase has even been found to convert host RNA into 5′-ppp dsRNA to induce IFN1 expression (266). This raises an intriguing possibility that induction of IFN1 may actually benefit some viruses under certain circumstances despite IFN1 signaling negatively regulating viral replication.

The viral RNA levels and localization throughout the viral life cycle might also play an important role in immune evasion (267). Control of viral RNA levels by viral exoribonucleases in particular illustrates the complicated balance between viral production and immune evasion for optimal viral propagation, as has found to be the case for arenaviral nucleoproteins (NPs) (268, 269) and non-structural proteins found in coronaviruses (270, 271). Finally, viral infection has the capability to disrupt processes of the cell's basic functions, such as transcription and translation, thereby affecting viral replication and immune signaling in complicated ways (258).

**Figure 6:**
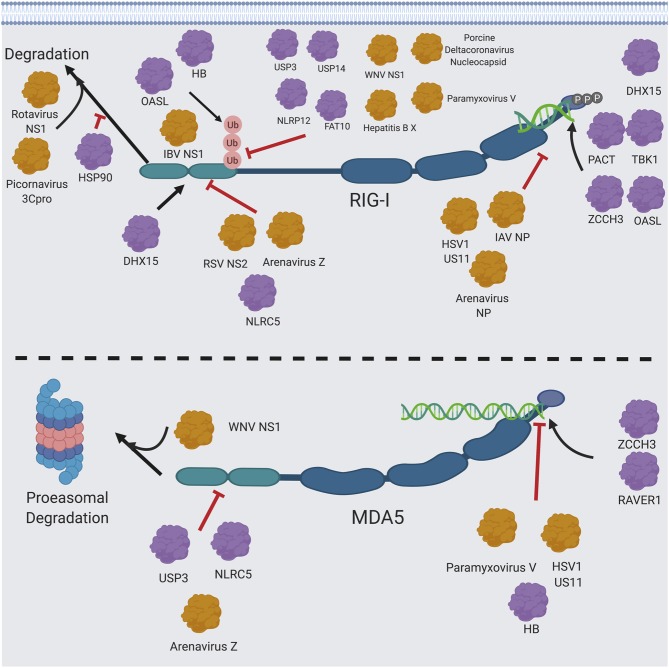
RIG-I/MDA5 interactions with host and cellular proteins. Host proteins (shown in purple) and viral proteins (shown in orange) that modulate RIG-I and MDA5 signaling are shown. Figure created using BioRender software.

One of the most significant ways viruses modulate RIG-I and MDA5 signaling is through their viral proteins (272) (Figure 6). The respiratory syncytial virus (RSV) non-structural protein (NS2) protein and the Z matrix proteins of pathogenic arenaviruses interact with the RIG-I CARD domains to block its interaction with MAVS (273, 274). The HSV1 deamidase UL37 specifically targets RIG-I through its helicase domain, abrogating its ability to bind to RNA (275). The IAV polymerase components also interact directly with RIG-I (276), though their biological significance has yet to be determined as they don't significantly affect IFN1 production. On the other hand, RNA binding appears to be an important bridge between the interaction of RIG-I with other viral proteins, as the nucleoproteins (NPs) of IAV (276) and arenaviruses (277, 278) both interact with RIG-I through viral RNA. The NS1 protein of rotaviruses targets RIG-I for degradation that is independent of proteasomes (279). The V protein of paramyxoviruses inhibits MDA5 (40) by targeting a unique feature of the ATP binding pocket in MDA5 (280) and by inhibiting MDA5 CARD dephosphorylation (93), but can also inhibit RIG-I by interacting with the CARD domain to prevent its ubiquitination by TRIM25 (281). Finally, the US11 protein of HSV1 (282) and the arenaviral Z matrix proteins (274) directly interact with and inhibit RIG-I and MDA5 in a similar fashion. There are also many other viral proteins that can regulate proteins in the RIG-I and MDA5 pathways, which have been discussed in detail elsewhere (44, 53, 59, 96, 257, 283).

## Modulations of RIG-I and MDA5 Functions by Their Post-translational Modifications and/or by Other Viral or Cellular Proteins

It is important to consider the different regulatory mechanisms of RIG-I and MDA5 when considering their different functionalities (Figures 4, 6). One of the key differences between these proteins is in their post-translation modifications (96). Ubiquitination of RIG-I is necessary for its activation (118) and is a point of negative regulation by host proteins (117, 284, 285), viral proteins (281, 286, 287) and ubiquitin mimics (288) as well as positively regulated by influenza B NS1 protein (289) and another ubiquitin mimic (290). On the contrary, MDA5 is more well-known to be negatively regulated by ubiquitination (291), with positive regulation by K63 ubiquitination being more controversial. While the deubiquitinase USP3 inhibits MDA5 as well as RIG-I, it is thought that this may be due to USP3 directly binding the MDA5 CARD domain to prevent RNA filamentation (284). This raises the question of how RIG-I can maintain its stability outside of the proteasome, as ubiquitination at other lysine residues in RIG-I besides K172 induces proteasomal degradation (291–293). This proteasomal degradation may be mediated by a p62 autophagic complex that associates with LRRC25/ISG15 (294) and SQSTM1 (295) and also mediates mitophagy and downregulation of MAVS signaling during measles virus infection (296).

One key observation is that, while both RIG-I and MDA5 are cleaved during picornavirus infection, this cleavage is mediated by the viral proteinase 3C^pro^ (297) and is independent of the proteasome (298) for RIG-I, whereas it is mediated by cellular caspases and the proteasome for MDA5 (299). MDA5 is also cleaved by caspases during apoptosis (4), though it hasn't been shown whether this is mediated by MDA5's ubiquitination sites. The ubiquitin linkage site may be a determinate of function, as the ubiquitin ligases RNF122 (300) and STUB1 (293, 301) have been shown to negatively regulate RIG-I catalyzed K48-linked ubiquitination as opposed to the known K63-linked ubiquitination at the K172, K849 and K851 activating sites, and RNF125 has also been proposed to K48 ubiquitinate RIG-I (291) (though it hasn't been shown directly) (59). TRIM40 has also been shown to negatively regulate RIG-I and MDA5 by K27 and K48 ubiquitination (123).

Substantiating the possibility that K63 ubiquitination on RIG-I may be functionally distinct from its other ubiquitination sites by protecting it from degradation is the finding that the NS1 protein of West Nile virus (WNV) targets both RIG-I and MDA5 for degradation by proteasomes. Additionally, NS1 inhibited K63 ubiquitination of RIG-I, but MDA5 was not found to be K63 ubiquitinated (126). Heat shock protein 90-alpha (HSP90) has been found to protect RIG-I from proteasomal degradation, but it is unknown which type of ubiquitination that is inhibited by HSP90 (302). Taken together, the experimental evidence suggests that RIG-I may be protected from proteasome degradation despite its activating ubiquitin moieties (52). This warrants further studies for mechanistic elucidation.

RIG-I and MDA5 additionally interact with different cellular co-factors, contributing to their differential regulations of function. RIG-I is well-known for being potentiated by proteins that also bind dsRNA, such as (PACT) (303, 304), which was first discovered as a protein activator of PKR, the serine/threonine-protein kinase 1 (TBK1) (305–309) and the oligoadenylate synthetase L (OASL) (310). PACT in particular has some functional similarities to RIG-I, as they each contain three distinct RNA binding domains (311) and interact with many of the same cellular co-factors, such as PKR (312) and Dicer (312, 313). Because of the important role of PACT in augmenting RIG-I function, it is a prime target for inhibition of RIG-I signaling by several viral proteins from diverse families of viruses (314–316), the molecular mechanisms of PACT inhibition by these viral proteins can vary and still need to be characterized in detail in future studies. Similarly, the host ribonucleoprotein RAVER1 can increase affinity of MDA5 for dsRNA (317), and the zinc-finger protein ZCCHC3 has recently been found do so for both RIG-I and MDA5 (125) in similar mechanisms to the other known RNA-binding proteins. On the contrary, the human hemoglobin subunit beta (HB) has recently been suggested to decrease MDA5 signaling by competing for long dsRNA, while HB can enhance RIG-I signaling by increasing K63 ubiquitination on RIG-I (318).

Several host factors interacting with RIG-I and MDA5 do so by yet undescribed mechanisms. PKR [which is also activated by PACT (319, 320) and is sequestered by the cellular helicase DHX36 protein to form stress granules (321, 322) along with RIG-I (323, 324) and TRIM25 (324)] appears to have a novel and yet uncharacterized function in enhancing MDA5-dependent MAVS signaling that is dependent on the kinase activity of PKR (325). Additionally, the porcine Interferon-Inducible Oligoadenylate Synthetase-like protein (pOASL) has also been found to interact with and inhibit MDA5 by an unknown mechanism (326).

The RIG-I CARD domain interacts with MAVS to induce interferon signaling, so proteins that disrupt this interaction [as it has been proposed for the Atg5 and Atg12 autophagy proteins (59)] can specifically inhibit RIG-I signaling. However, other cellular proteins, such as the complement protein gC1qR (327) and TARBP2 (328) that interact directly with MAVS, inhibit both RIG-I and MDA5. Lactate and hexokinase have also recently been found to inhibit RIG-I and MDA5 by interacting with MAVS, which may be significant in explaining the interplay between metabolism and immune signaling as glycolysis was found to be greatly decreased upon RLR signaling (329). Likewise, cellular proteins, such as NLRC5 (330) that interacts with the RIG-I and MDA5 CARD domains have been shown to block interaction of both RIG-I and MDA5 with MAVS. Contrarily, DHX15 has been identified as a RIG-I cofactor that interacts with the RIG-I CARD domains and with PAMP (dsRNA), thereby increasing RIG-I ATPase activity (331). Additionally, ADP-ribosylation factor proteins can block RIG-I and MDA5 from interacting with PAMPs and thereby inhibit their activation (332, 333). Lastly, the green tea molecule EGCG has also been shown to inhibit the ATPase function of RIG-I (334). The similarities and differences between RIG-I and MDA5 modulations and signaling are complex and will need to be elucidated further in future studies.

## Evolution and Speciation of RIG-I/MDA5 and RIG-I/MDA5-like Proteins

**Figure 7:**
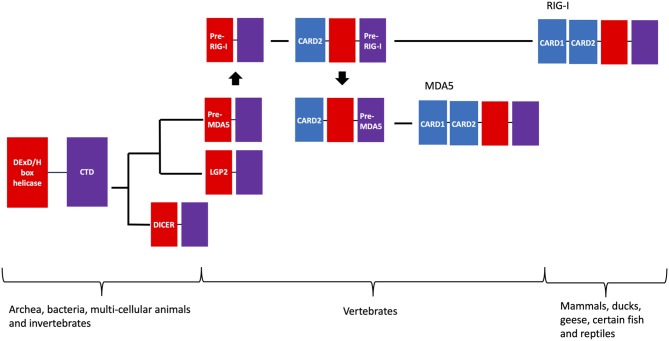
Evolutionary timeline of RIG-I, MDA5, and other related DExD/H-box helicases. The evolutionary tract of RIG-I, MDA5, and related DExD/H-box helicases are shown as a phylogenetic tree, along with their lowest level of biological taxonomy that these proteins are found in present day. In short, the precursor of the MDA5 helicase-CTD likely originated from a common ancestor with the precursor for LGP2, which was then duplicated to create the helicase-CTD precursor of RIG-I in the common ancestor of vertebrates. CARD2 was then grafted onto the helicase-CTD protein, and this protein was duplicated to create the CARD2-helicase-CTD precursor of MDA5. Finally, CARD1 was grafted onto these proteins in separate events, forming the modern-day RIG-I and MDA5.

Despite their structural and mechanistic differences, it is important to emphasize that existing phylogenetic analysis indicates that RIG-I and MDA5 come from a common origin that is also shared among several other protein families (Figure 7). The linkage of the helicase and DExD/H box protein appear to be ancient, as orthologs of these proteins are found in the Archaea kingdom (335, 336). MDA5 orthologs are found in most vertebrates (184), while RIG-I orthologs are only found in mammals, ducks, geese and some selected fish and reptiles (184, 337–343) (Figures 2, 7).

It is therefore likely that MDA5 evolved first, perhaps from a common ancestor with the closely related LGP2 helicase family (184), which is structural similar to RIG-I and MDA5 but lacks the CARD domains at its N terminus (185). LGP2 orthologs are also only found in vertebrates while the next closest related family of proteins (Dicer) are more ancient proteins. It has therefore been proposed that the RIG-I helicase-DExD/H complex may have been duplicated from MDA5 in the common ancestor of vertebrates (184). The association of the two CARD domains appears to have followed, as individual CARD domains are found in a variety of vertebrates that also encode caspases (344, 345), but only RIG-I, MDA5, and certain members of the Nacht family of NTPases (346) have two CARD domains. Phylogenetic analysis has shown that the helicase-DExD/H and CARD2 have strong co-evolution history (347, 348), while CARD1 has evolved more independently (184). CARD2 appears to have been grafted onto the RIG-I helicase-DExD/H complex first, with the CARD2-MDA5 being duplicated from this event. Finally, CARD1 was grafted onto the CARD2-helicase-DExD/H complex in separate events for RIG-I and MDA5 (184). In mammals, positive selection can be seen in the flexible hinge region connecting the CARD domains to the helicase in RIG-I and MDA5. RIG-I contains an additional site of positive selection within the Hel1 structural motif (N421), while most of the unique positive selection sites for MDA5 are in regions specific to it, including a 29 amino acid insertion in Hel2 (349).

While RIG-I and MDA5 may both originate from common ancestors of vertebrates, there is increasing evidence to suggest that proteins with similar functions may have evolved separately in other species from ancient helicase-DExD/H proteins, implicating RNA-mediated defense responses as a potentially universal biological function. A RIG-I homolog has recently been found in a planarian that is able to activate downstream inflammatory genes in the absence of the traditional CARD domains (350), and a similar homolog in *Caenorhabditis elegans* has been proposed to mediate anti-viral RNAi by complexing with Dicer and catalyzing their translocation on the viral genome (351). Additionally, insects have been found to primarily respond to RNA viruses by RNAi mediated by Dicer proteins (352). Dicer may potentially mediate dsRNA-activated anti-viral signaling pathways that is independent of RNAi pathways, as has been found to be the case for the expanded CAG-repeat dsRNA (353). Pattern recognition receptors (PRRs) that respond to viral RNA have not yet been found outside of the animal kingdom, as RLR-like proteins in prokaryotes do not have CARD domains and the PRRs in plants found so far are surface-receptor kinases that respond to external molecular elements of bacteria (354) [similar to the mammalian toll-like receptors (TLRs)]. However, RNA silencing has been demonstrated to be an important anti-viral strategy in plants (354, 355) and certain *Arabidopsis* mutants appear to be more susceptible to infection by RNA viruses (356).

RIG-I (357) and MDA5 (357, 358) are known to influence antiviral signaling in zebrafish (*Danio rerio*) and other fish species (357, 359–361) through the canonical MAVS signaling pathway. Fish RIG-I like receptors (RLRs) have been shown to be regulated by the expression of alternate splicing isoforms (358, 362), which have also been found to occur with a dominant-negative splice variant of the human RIG-I (363). RIG-I and MDA5 have also been found to participate in anti-viral signaling in ducks (364–367) and geese (340, 368, 369), and MDA5 alone in chickens (370–372) and other birds (373). The observation across species of RLR's performing compensatory mechanisms when a function or a pathway protein is absent is reiterated in birds, as MDA5 has been found to sense short and long dsRNA in chickens (372) and in the Chinese shrew (374), both of which lack RIG-I. Additionally, TRIM25 activates RIG-I in ducks (364) and in the Chinese goose (375) in the absence of the K172 activating ubiquitin binding site that is conserved in primates and some rodents (364). Finally, the rainbow trout (*Oncorhynchus mykiss*) has been found to express a LGP2 variant in addition to the canonical LGP2 that contains an incomplete C-terminal domain of RIG-I (376). The differential presence of PRRs may also influence viral evolution. A mutation in the IAV polymerase subunit PB2 found in avian-adapted H1N1 strains decreases the inhibition of human RIG-I function by IAV nucleoproteins, which may indicate a differential selective pressure for viruses that propagate in species that don't contain RIG-I (377). The evolutionary pattern and compensatory mechanisms of RLRs across species implicate them as critical for anti-viral function, and that evolutionary forces drive the available pathway proteins to meet these functional needs. Future studies need to be done to further differentiate RLR function among the different species, as this will provide critical information concerning the various methods of disease control by targeting the pathogen by these important host proteins.

There is also increasing evidence for other RNA-sensing DExD/H helicases serving important roles in anti-pathogen immune sensing, which have recently been reviewed elsewhere (187). Some RNA helicase (DDX) proteins appear to serve as complex proteins upon interacting with viral RNA. DDX3 is a well-known example, being suspected of being a transcription factor for IFN-β (378), associating with spliceosomes and the stress-induced p-bodies to influence mRNA splicing and decay, respectively (322, 378), and interacting with the MAVS complex during viral infection conditions (378, 379). In particular, DDX3 associating with MAVS has been found to be important for anti-viral control against several viruses (378–380), and since the two DDX3 homologs are found on the X and Y chromosomes, they may contribute to immunological differences between genders (381). This is a repeated theme, as DHX9 (382), DHX15 (383), and a complex consisting of DDX1/DDX21/DHX36 (384) have also been found to associate with the MAVS complex to enhance IFN1 signaling, while DHX33 interacts with MAVS independently of viral infection (385). DDX proteins can also activate other proteins in the IRF pathway. Multiple DDX proteins can interact with IKKε, with DDX3 being phosphorylated by IKKε to induce IRF3 interaction with the TBK1-IKKε complex (378), and DDX19 blocking this interaction to inhibit IFN1 signaling (386). Similar control mechanisms have been demonstrated for DDX3 interacting with viral proteins. For example, DDX3 has recently been found to associate with arenaviral NPs to increase viral RNA synthesis and IFN1 expression (387). Additionally, the NP of the 1918 H1N1 IAV pandemic strain has been shown to target DDX3 for degradation as a potential mechanism of virulence (388). DHX15 (389) and DHX33 have also been found to activate NFκB and MAPK signaling pathways. Finally, DDX60 has been shown to act as a cofactor for RIG-I (390, 391) and DHX29 for MDA5 (392). Taken altogether, these cellular proteins have likely evolved to regulate RIG-I and MDA5 signaling from their common DExD/H helicase predecessors.

## Summary and Future Outlooks

As our capacity to study the molecular mechanisms and to purposefully modulate immune responses increases in specificity, so will our needs to characterize the differences between related immune signaling proteins. The concept of personalized medicine derives from the idea that we can therapeutically intervene in a situation that is designed around the individual's unique characteristics. While this is an achievable realm of medicine in the future, an immediate step is to determine the functions of some critical proteins, such as the RIG-I and MDA5 of the innate immune arm. Examining their structural and functional similarities and differences at multiple levels will allow for a deeper level of appreciation of these proteins, which may be exploited therapeutically to differentially modulate RIG-I and MDA5 signalings by different RNA ligands (43, 191, 393, 394) or other pharmaceutical compounds (395) toward the goal of achieving personalized medicine.

## Author Contributions

MB and HL contributed to the literature review and writing of the manuscript. MB prepared all figures with inputs from HL.

### Conflict of Interest Statement

The authors declare that the research was conducted in the absence of any commercial or financial relationships that could be construed as a potential conflict of interest.

## References

[1] Sun YW RIG-I, a human homolog gene of RNA helicase, is induced by retinoic acid during the differentiation of acute promyelocytic leukemia cell. Biochem Biophys Res Commun. (1997) 292:274–9.

[2] Imaizumi T Aratani S Nakajima T Carlson M Matsumiya T Tanji K. Retinoic acid-inducible gene-I is induced in endothelial cells by LPS and regulates expression of COX-2. Biochem Biophys Res Commun. (2002) 292:274–9. 10.1006/BBRC.2002.6650 11890704

[3] Kang D Gopalkrishnan RV Wu Q Jankowsky E Pyle AM Fisher PB. mda-5: an interferon-inducible putative RNA helicase with double-stranded RNA-dependent ATPase activity and melanoma growth-suppressive properties. Proc Natl Acad Sci USA. (2002) 99:637–42. 10.1073/PNAS.022637199 11805321 PMC117358

[4] Kovacsovics M Martinon F Micheau O Bodmer J-L Hofmann K Tschopp J. Overexpression of helicard, a CARD-containing helicase cleaved during apoptosis, accelerates DNA degradation. Curr Biol. (2002) 12:838–43. 10.1016/S0960-9822(02)00842-4 12015121

[5] Randall RE Goodbourn S. Interferons and viruses: an interplay between induction, signalling, antiviral responses and virus countermeasures. J Gen Virol. (2008) 89:1–47. 10.1099/vir.0.83391-0 18089727

[6] Jacobs JL Coyne CB. Mechanisms of MAVS regulation at the mitochondrial membrane. J Mol Biol. (2013) 425:5009–19. 10.1016/j.jmb.2013.10.007 24120683 PMC4562275

[7] Vazquez C Horner SM. MAVS coordination of antiviral innate immunity. J Virol. (2015) 89:6974–7. 10.1128/JVI.01918-14 25948741 PMC4473567

[8] Tan P He L Cui J Qian C Cao X Lin M. Assembly of the WHIP-TRIM14-PPP6C mitochondrial complex promotes RIG-I-mediated antiviral signaling. Mol Cell. (2017) 68:293–307.e5. 10.1016/J.MOLCEL.2017.09.035 29053956

[9] Kouwaki T Okamoto M Tsukamoto H Fukushima Y Matsumoto M Seya T. Zyxin stabilizes RIG-I and MAVS interactions and promotes type I interferon response. Sci Rep. (2017) 7:11905. 10.1038/s41598-017-12224-7 28928438 PMC5605516

[10] Fitzgerald KA McWhirter SM Faia KL Rowe DC Latz E Golenbock DT. IKKε and TBK1 are essential components of the IRF3 signaling pathway. Nat Immunol. (2003) 4:491–6. 10.1038/ni921 12692549

[11] Sharma S TenOever BR Grandvaux N Zhou G-P Lin R Hiscott J. Triggering the interferon antiviral response through an IKK-related pathway. Science. (2003) 300:1148–51. 10.1126/science.1081315 12702806

[12] tenOever BR Ng S-L Chua MA McWhirter SM Garcia-Sastre A Maniatis T. Multiple functions of the IKK-related kinase IKKe in interferon-mediated antiviral immunity. Science. (2007) 315:1274–8. 10.1126/science.1138527 17332413

[13] Paz S Sun Q Nakhaei P Romieu-Mourez R Goubau D Julkunen I. Induction of IRF-3 and IRF-7 phosphorylation following activation of the RIG-I pathway. Cell Mol Biol. (2006) 52:17–28. 16914100

[14] Ning S Pagano JS Barber GN. IRF7: activation, regulation, modification and function. Genes Immun. (2011) 12:399–414. 10.1038/gene.2011.21 21490621 PMC4437765

[15] Mingzhu Zhu G Fang T Li S Meng K. Activity of IFN regulatory factor 3 controls nuclear import and DNA-binding bipartite nuclear localization signal. J Immunol. (2015) 195:289–97. 10.4049/jimmunol.1500232 25994966

[16] Newton K Dixit VM. Signaling in innate immunity and inflammation. Cold Spring Harb Perspect Biol. (2012) 4:a006049. 10.1101/cshperspect.a006049 22296764 PMC3282411

[17] Kell AM Gale M Jr RIG-I in RNA virus recognition. Virology. (2015) 479–80:110–21. 10.1016/j.virol.2015.02.017 PMC4424084 25749629

[18] Kato H Takeuchi O Sato S Yoneyama M Yamamoto M Matsui K. Differential roles of MDA5 and RIG-I helicases in the recognition of RNA viruses. Nature. (2006) 441:101–5. 10.1038/nature04734 16625202

[19] Weber-Gerlach M Weber F. Standing on three legs: antiviral activities of RIG-I against influenza viruses. Curr Opin Immunol. (2016) 42:71–5. 10.1016/J.COI.2016.05.016 27318973

[20] Spengler JR Patel JR Chakrabarti AK Zivcec M García-Sastre A Spiropoulou CF. RIG-I mediates an antiviral response to crimean-congo hemorrhagic fever virus. J Virol. (2015) 89:10219–29. 10.1128/JVI.01643-15 26223644 PMC4580164

[21] Yamada S Shimojima M Narita R Tsukamoto Y Kato H Saijo M. RIG-I-like receptor and toll-like receptor signaling pathways cause aberrant production of inflammatory cytokines/chemokines in a severe fever with thrombocytopenia syndrome virus infection mouse model. J Virol. (2018) 92:e02246–17. 10.1128/JVI.02246-17 29643242 PMC6002725

[22] Spiropoulou CF Ranjan P Pearce MB Sealy TK Albariño CG Gangappa S. RIG-I activation inhibits ebolavirus replication. Virology. (2009) 392:11–5. 10.1016/J.VIROL.2009.06.032 19628240

[23] Furr SR Moerdyk-Schauwecker M Grdzelishvili VZ Marriott I. RIG-I mediates nonsegmented negative-sense RNA virus-induced inflammatory immune responses of primary human astrocytes. Glia. (2010) 58:1620–9. 10.1002/glia.21034 20578054 PMC2946392

[24] Loo Y-M Fornek J Crochet N Bajwa G Perwitasari O Martinez-Sobrido L. Distinct RIG-I and MDA5 signaling by RNA viruses in innate immunity. J Virol. (2008) 82:335–45. 10.1128/JVI.01080-07 17942531 PMC2224404

[25] Deddouche S Goubau D Rehwinkel J Chakravarty P Begum S Maillard PV. Identification of an LGP2-associated MDA5 agonist in picornavirus-infected cells. Elife. (2014) 3:e1535. 10.7554/eLife.01535 24550253 PMC3967861

[26] Luo R Xiao S Jiang Y Jin H Wang D Liu M. Porcine reproductive and respiratory syndrome virus (PRRSV) suppresses interferon-β production by interfering with the RIG-I signaling pathway. Mol Immunol. (2008) 45:2839–46. 10.1016/J.MOLIMM.2008.01.028 18336912 PMC7112510

[27] van Kasteren PB Beugeling C Ninaber DK Frias-Staheli N van Boheemen S Garcia-Sastre A. Arterivirus and nairovirus ovarian tumor domain-containing deubiquitinases target activated RIG-I to control innate immune signaling. J Virol. (2012) 86:773–85. 10.1128/JVI.06277-11 22072774 PMC3255818

[28] Zhang Z Filzmayer C Ni Y Sültmann H Mutz P Hiet M-S. Hepatitis D virus replication is sensed by MDA5 and induces IFN-β/λ responses in hepatocytes. J Hepatol. (2018) 69:25–35. 10.1016/j.jhep.2018.02.021 29524530

[29] Zhao Y Ye X Dunker W Song Y Karijolich J. RIG-I like receptor sensing of host RNAs facilitates the cell-intrinsic immune response to KSHV infection. Nat Commun. (2018) 9:4841. 10.1038/s41467-018-07314-7 30451863 PMC6242832

[30] Ye W Chew M Hou J Lai F Leopold SJ Loo HL. Microvesicles from malaria-infected red blood cells activate natural killer cells via MDA5 pathway. PLoS Pathog. (2018) 14:e1007298. 10.1371/journal.ppat.1007298 30286211 PMC6171940

[31] Qin C-F Zhao H Liu Z-Y Jiang T Deng Y-Q Yu X-D Retinoic acid inducible gene-I and melanoma differentiation-associated gene 5 are induced but not essential for dengue virus induced type I interferon response. Mol Biol Rep. (2011) 38:3867–73. 10.1007/s11033-010-0502-7 21113677

[32] Nasirudeen AMA Wong HH Thien P Xu S Lam K-P Liu DX. RIG-I, MDA5 and TLR3 synergistically play an important role in restriction of dengue virus infection. PLoS Negl Trop Dis. (2011) 5:e926. 10.1371/journal.pntd.0000926 21245912 PMC3014945

[33] Fredericksen BL Keller BC Fornek J Katze MG Gale M Establishment and maintenance of the innate antiviral response to West Nile virus involves both RIG-I and MDA5 signaling through IPS-1†. J Virol. (2008) 82:609–16. 10.1128/JVI.01305-07 17977974 PMC2224571

[34] Errett JS Suthar MS McMillan A Diamond MS Gale M. The essential, nonredundant roles of RIG-I and MDA5 in detecting and controlling West Nile virus infection. J Virol. (2013) 87:11416–25. 10.1128/JVI.01488-13 23966395 PMC3807316

[35] Sanchez David RY Combredet C Sismeiro O Dillies M-A Jagla B Coppée J-Y. Comparative analysis of viral RNA signatures on different RIG-I-like receptors. Elife. (2016) 5:e11275. 10.7554/eLife.11275 27011352 PMC4841775

[36] Gitlin L Benoit L Song C Cella M Gilfillan S Holtzman MJ. Melanoma Differentiation-Associated Gene 5 (MDA5) is involved in the innate immune response to paramyxoviridae infection *in vivo*. PLoS Pathog. (2010) 6:e1000734. 10.1371/journal.ppat.1000734 20107606 PMC2809771

[37] Grandvaux N Guan X Yoboua F Zucchini N Fink K Doyon P. Sustained activation of interferon regulatory factor 3 during infection by paramyxoviruses requires MDA5. J Innate Immun. (2014) 6:650–62. 10.1159/000360764 24800889 PMC4846353

[38] Kim W-K Jain D Sánchez MD Koziol-White CJ Matthews K Ge MQ. Deficiency of melanoma differentiation-associated protein 5 results in exacerbated chronic postviral lung inflammation. Am J Respir Crit Care Med. (2014) 189:437–48. 10.1164/rccm.201307-1338OC 24417465 PMC3977719

[39] Broquet AH Hirata Y McAllister CS Kagnoff MF. RIG-I/MDA5/MAVS are required to signal a protective IFN response in rotavirus-infected intestinal epithelium. J Immunol. (2011) 186:1618–26. 10.4049/JIMMUNOL.1002862 21187438

[40] Yoneyama M Kikuchi M Matsumoto K Imaizumi T Miyagishi M Taira K. Shared and unique functions of the DExD/H-box helicases RIG-I, MDA5, and LGP2 in antiviral innate immunity. J Immunol. (2005) 175:2851–8. 10.4049/JIMMUNOL.175.5.2851 16116171

[41] Kato H Sato S Yoneyama M Yamamoto M Uematsu S Matsui K. Cell type-specific involvement of RIG-I in antiviral response. Immunity. (2005) 23:19–28. 10.1016/J.IMMUNI.2005.04.010 16039576

[42] Wang Y Zhang H-X Sun Y-P Liu Z-X Liu X-S Wang L Rig-I –/– mice develop colitis associated with downregulation of Gαi2. Cell Res. (2007) 17:858–68. 10.1038/cr.2007.81 17893708

[43] Kasumba DM Grandvaux N Therapeutic targeting of RIG-I and MDA5 might not lead to the same Rome. Trends Pharmacol Sci. (2018) 40:116–27. 10.1016/j.tips.2018.12.003 30606502 PMC7112877

[44] Ng CS Kato H Fujita T. Fueling type I interferonopathies: regulation and function of type I interferon antiviral responses. J Interf Cytokine Res. (2019). 10.1089/jir.2019.0037. [Epub ahead of print]. 30897023

[45] Peckham D Scambler T Savic S McDermott MF. The burgeoning field of innate immune-mediated disease and autoinflammation. J Pathol. (2017) 241:123–39. 10.1002/path.4812 27682255

[46] Czerkies M Korwek Z Prus W Kochanczyk M Jaruszewicz-Błonska J Tudelska K. Cell fate in antiviral response arises in the crosstalk of IRF, NF-κB and JAK/STAT pathways. Nat Commun. (2018) 9:493. 10.1038/s41467-017-02640-8 29402958 PMC5799375

[47] Yoboua F Martel A Duval A Mukawera E Grandvaux N. Respiratory syncytial virus-mediated NF-kappa B p65 phosphorylation at serine 536 is dependent on RIG-I, TRAF6, and IKK beta. J Virol. (2010) 84:7267–77. 10.1128/JVI.00142-10 20410276 PMC2898247

[48] Rückle A Haasbach E Julkunen I Planz O Ehrhardt C Ludwig S. The NS1 protein of influenza A virus blocks RIG-I-mediated activation of the noncanonical NF-κB pathway and p52/RelB-dependent gene expression in lung epithelial cells. J Virol. (2012) 86:10211–7. 10.1128/JVI.00323-12 22787206 PMC3446553

[49] Bertin J Guo Y Wang L Srinivasula SM Jacobson MD Poyet JL. CARD9 is a novel caspase recruitment domain-containing protein that interacts with BCL10/CLAP and activates NF-kappa B. J Biol Chem. (2000) 275:41082–6. 10.1074/jbc.C000726200 11053425

[50] Poeck H Bscheider M Gross O Finger K Roth S Rebsamen M Recognition of RNA virus by RIG-I results in activation of CARD9 and inflammasome signaling for interleukin 1β production. Nat Immunol. (2010) 11:63–9. 10.1038/ni.1824 19915568

[51] Lee N-R Kim H-I Choi M-S Yi C-M Inn K-S. Regulation of MDA5-MAVS antiviral signaling axis by TRIM25 through TRAF6-mediated NF-κB activation. Mol Cells. (2015) 38:759–64. 10.14348/molcells.2015.0047 26299329 PMC4588718

[52] Martín-Vicente M Medrano LM Resino S García-Sastre A Martínez I. TRIM25 in the regulation of the antiviral innate immunity. Front Immunol. (2017) 8:1187. 10.3389/fimmu.2017.01187 29018447 PMC5614919

[53] Loo Y-M Gale M Jr. Immune signaling by RIG-I-like receptors. Immunity. (2011) 34:680–92. 10.1016/j.immuni.2011.05.003 21616437 PMC3177755

[54] Song M-S Rossi JJ. Molecular mechanisms of Dicer: endonuclease and enzymatic activity. Biochem J. (2017) 474:1603–18. 10.1042/BCJ20160759 28473628 PMC5415849

[55] van der Veen AG Maillard PV Schmidt JM Lee SA Deddouche-Grass S Borg A. The RIG-I-like receptor LGP2 inhibits Dicer-dependent processing of long double-stranded RNA and blocks RNA interference in mammalian cells. EMBO J. (2018) 37:e97479. 10.15252/embj.201797479 29351913 PMC5813259

[56] Takahashi T Nakano Y Onomoto K Yoneyama M Ui-Tei K. Virus sensor RIG-I represses RNA interference by interacting with TRBP through LGP2 in mammalian cells. Genes. (2018) 9:E511. 10.3390/genes9100511 30347765 PMC6210652

[57] Moresco EMY Beutler B. LGP2: positive about viral sensing. Proc Natl Acad Sci USA. (2010) 107:1261–2. 10.1073/pnas.0914011107 20133887 PMC2824419

[58] Rodriguez KR Bruns AM Horvath CM. MDA5 and LGP2: accomplices and antagonists of antiviral signal transduction. J Virol. (2014) 88:8194–200. 10.1128/JVI.00640-14 24850739 PMC4135949

[59] Quicke KM Diamond MS Suthar MS. Negative regulators of the RIG-I-like receptor signaling pathway. Eur J Immunol. (2017) 47:615–28. 10.1002/eji.201646484 28295214 PMC5554756

[60] Uhlen M Fagerberg L Hallstrom BM Lindskog C Oksvold P Mardinoglu A. Tissue-based map of the human proteome. Science. (2015) 347:1260419. 10.1126/science.1260419 25613900

[61] Devarkar SC Wang C Miller MT Ramanathan A Jiang F Khan AG. Structural basis for m7G recognition and 2'-O-methyl discrimination in capped RNAs by the innate immune receptor RIG-I. Proc Natl Acad Sci USA. (2016) 113:596–601. 10.1073/pnas.1515152113 26733676 PMC4725518

[62] Wu B Peisley A Richards C Yao H Zeng X Lin C. Structural basis for dsRNA recognition, filament formation, and antiviral signal activation by MDA5. Cell. (2013) 152:276–89. 10.1016/j.cell.2012.11.048 23273991

[63] Wu B Peisley A Tetrault D Li Z Egelman EHH Magor KEE. Molecular imprinting as a signal-activation mechanism of the viral RNA sensor RIG-I. Mol Cell. (2014) 55:511–23. 10.1016/j.molcel.2014.06.010 25018021 PMC4142144

[64] Rawling DC Pyle AM. Parts, assembly and operation of the RIG-I family of motors. Curr Opin Struct Biol. (2014) 25:25–33. 10.1016/J.SBI.2013.11.011 24878341 PMC4070197

[65] Yoneyama M Kikuchi M Natsukawa T Shinobu N Imaizumi T Miyagishi M. The RNA helicase RIG-I has an essential function in double-stranded RNA-induced innate antiviral responses. Nat Immunol. (2004) 5:730–7. 10.1038/ni1087 15208624

[66] Cui S Eisenächer K Kirchhofer A Brzózka K Lammens A Lammens K. The C-terminal regulatory domain is the RNA 5'-triphosphate sensor of RIG-I. Mol Cell. (2008) 29:169–79. 10.1016/j.molcel.2007.10.032 18243112

[67] Hornung V Ellegast J Kim S Brzózka K Jung A Kato H. 5′-Triphosphate RNA is the ligand for RIG-I. Science. (2006) 314:994–7. 10.1126/science.1132505 17038590

[68] Pichlmair A Schulz O Tan CP Naslund TI Liljestrom P Weber F. RIG-I-mediated antiviral responses to single-stranded RNA bearing 5'-Phosphates. Science. (2006) 314:997–1001. 10.1126/science.1132998 17038589

[69] Takahasi K Yoneyama M Nishihori T Hirai R Kumeta H Narita R. Nonself RNA-sensing mechanism of RIG-I helicase and activation of antiviral immune responses. Mol Cell. (2008) 29:428–40. 10.1016/j.molcel.2007.11.028 18242112

[70] Myong S Cui S Cornish PV Kirchhofer A Gack MU Jung JU. Cytosolic viral sensor RIG-I is a 5'-Triphosphate–dependent translocase on double-stranded RNA. Science. (2009) 323:1070–4. 10.1126/science.1168352 19119185 PMC3567915

[71] Lu C Xu H Ranjith-Kumar CT Brooks MT Hou TY Hu F. The structural basis of 5' triphosphate double-stranded RNA recognition by RIG-I C-terminal domain. Structure. (2010) 18:1032–43. 10.1016/j.str.2010.05.007 20637642 PMC2919622

[72] Rehwinkel J Tan CP Goubau D Schulz O Pichlmair A Bier K. RIG-I detects viral genomic RNA during negative-strand RNA virus infection. Cell. (2010) 140:397–408. 10.1016/j.cell.2010.01.020 20144762

[73] Wang Y Ludwig J Schuberth C Goldeck M Schlee M Li H. Structural and functional insights into 5'-ppp RNA pattern recognition by the innate immune receptor RIG-I. Nat Struct Mol Biol. (2010) 17:781–7. 10.1038/nsmb.1863 20581823 PMC3744876

[74] Vela A Fedorova O Ding SC Pyle AM. The thermodynamic basis for viral RNA detection by the RIG-I innate immune sensor. J Biol Chem. (2012) 287:42564–73. 10.1074/jbc.M112.385146 23055530 PMC3522258

[75] Anchisi S Guerra J Garcin D. RIG-I ATPase activity and discrimination of self-RNA versus non-self-RNA. MBio. (2015) 6:e02349–14. 10.1128/MBIO.02349-14 25736886 PMC4358010

[76] Kato H Takeuchi O Mikamo-Satoh E Hirai R Kawai T Matsushita K. Length-dependent recognition of doublestranded ribonucleic acids by retinoic acid – inducible gene-I and melanoma differentiation – associated gene 5. J Exp Med. (2008) 205:1601–10. 10.1084/jem.20080091 18591409 PMC2442638

[77] Pichlmair A Schulz O Tan C-P Rehwinkel J Kato H Takeuchi O. Activation of MDA5 requires higher-order RNA structures generated during virus infection. J Virol. (2009) 83:10761–9. 10.1128/JVI.00770-09 19656871 PMC2753146

[78] Feng Q Hato SV Langereis MA Zoll J Virgen-Slane R Peisley A. MDA5 detects the double-stranded RNA replicative form in picornavirus-infected cells. Cell Rep. (2012) 2:1187–96. 10.1016/j.celrep.2012.10.005 23142662 PMC7103987

[79] Triantafilou K Vakakis E Kar S Richer E Evans GL Triantafilou M. Visualisation of direct interaction of MDA5 and the dsRNA replicative intermediate form of positive strand RNA viruses. J Cell Sci. (2012) 125:4761–9. 10.1242/jcs.103887 22797917

[80] Kolakofsky D Kowalinski E Cusack S. A structure-based model of RIG-I activation. RNA. (2012) 18:2118–27. 10.1261/rna.035949.112 23118418 PMC3504664

[81] Luo D. Toward a crystal-clear view of the viral RNA sensing and response by RIG-I-like receptors. RNA Biol. (2014) 11:25–32. 10.4161/rna.27717 24457940 PMC3929420

[82] Reikine S Nguyen JB Modis Y. Pattern recognition and signaling mechanisms of RIG-I and MDA5. Front Immunol. (2014) 5:342. 10.3389/fimmu.2014.00342 25101084 PMC4107945

[83] Bruns AM Horvath CM. Antiviral RNA recognition and assembly by RLR family innate immune sensors. Cytokine Growth Factor Rev. (2014) 25:507–12. 10.1016/J.CYTOGFR.2014.07.006 25081315 PMC4252791

[84] Chiang C Gack MU. Post-translational control of intracellular pathogen sensing pathways. Trends Immunol. (2017) 38:39–52. 10.1016/J.IT.2016.10.008 27863906 PMC5580928

[85] Gee P Chua PK Gevorkyan J Klumpp K Najera I Swinney DC. Essential role of the N-terminal domain in the regulation of RIG-I ATPase activity. J Biol Chem. (2008) 283:9488–96. 10.1074/jbc.M706777200 18268020

[86] Kowalinski E Lunardi T McCarthy AA Louber J Brunel J Grigorov B. Structural basis for the activation of innate immune pattern-recognition receptor RIG-I by viral RNA. Cell. (2011) 147:423–35. 10.1016/j.cell.2011.09.039 22000019

[87] Feng M Ding Z Xu L Kong L Wang W Jiao S. Structural and biochemical studies of RIG-I antiviral signaling. Protein Cell. (2013) 4:142–54. 10.1007/s13238-012-2088-4 23264040 PMC4875364

[88] Nistal-Villán E Gack MU Martínez-Delgado G Maharaj NP Inn K-S Yang H. Negative role of RIG-I serine 8 phosphorylation in the regulation of interferon-beta production. J Biol Chem. (2010) 285:20252–61. 10.1074/jbc.M109.089912 20406818 PMC2888438

[89] Maharaj NP Wies E Stoll A Gack MU. Conventional protein kinase C-α (PKC-α) and PKC-β negatively regulate RIG-I antiviral signal transduction. J Virol. (2012) 86:1358–71. 10.1128/JVI.06543-11 22114345 PMC3264329

[90] Sun Z Ren H Liu Y Teeling JL Gu J. Phosphorylation of RIG-I by casein kinase II inhibits its antiviral response. J Virol. (2011) 85:1036–47. 10.1128/JVI.01734-10 21068236 PMC3020001

[91] Takashima K Oshiumi H Takaki H Matsumoto M Seya T. RIOK3-mediated phosphorylation of MDA5 interferes with its assembly and attenuates the innate immune response. Cell Rep. (2015) 11:192–200. 10.1016/j.celrep.2015.03.027 25865883

[92] Wies E Wang MK Maharaj NP Chen K Zhou S Finberg RW. Dephosphorylation of the RNA sensors RIG-I and MDA5 by the phosphatase PP1 is essential for innate immune signaling. Immunity. (2013) 38:437–49. 10.1016/j.immuni.2012.11.018 23499489 PMC3616631

[93] Davis ME Wang MK Rennick LJ Full F Gableske S Mesman AW. Antagonism of the phosphatase PP1 by the measles virus V protein is required for innate immune escape of MDA5. Cell Host Microbe. (2014) 16:19–30. 10.1016/J.CHOM.2014.06.007 25011105 PMC4120867

[94] Choi SJ Lee H-C Kim J-H Park SY Kim T-H Lee W-K. HDAC6 regulates cellular viral RNA sensing by deacetylation of RIG-I. EMBO J. (2016) 35:429–42. 10.15252/embj.201592586 26746851 PMC4755110

[95] Hilbert M Karow AR Klostermeier D. The mechanism of ATP-dependent RNA unwinding by DEAD box proteins. Biol Chem. (2009) 390:1237–50. 10.1515/BC.2009.135 19747077

[96] Gack MU. Mechanisms of RIG-I-like receptor activation and manipulation by viral pathogens. J Virol. (2014) 88:5213–6. 10.1128/JVI.03370-13 24623415 PMC4019093

[97] Weber F. The catcher in the RIG-I. Cytokine. (2015) 76:38–41. 10.1016/J.CYTO.2015.07.002 26168692

[98] Yoneyama M Onomoto K Jogi M Akaboshi T Fujita T. Viral RNA detection by RIG-I-like receptors. Curr Opin Immunol. (2015) 32:48–53. 10.1016/J.COI.2014.12.012 25594890

[99] Berke IC Modis Y. MDA5 cooperatively forms dimers and ATP-sensitive filaments upon binding double-stranded RNA. EMBO J. (2012) 31:1714–26. 10.1038/emboj.2012.19 22314235 PMC3321199

[100] Oshiumi H Matsumoto M Hatakeyama S Seya T. Riplet/RNF135, a RING finger protein, ubiquitinates RIG-I to promote interferon-beta induction during the early phase of viral infection. J Biol Chem. (2009) 284:807–17. 10.1074/jbc.M804259200 19017631

[101] Gao D Yang Y-K Wang R-P Zhou X Diao F-C Li M-D. REUL is a novel E3 ubiquitin ligase and stimulator of retinoic-acid-inducible gene-I. PLoS ONE. (2009) 4:e5760. 10.1371/journal.pone.0005760 19484123 PMC2684588

[102] Oshiumi H Miyashita M Matsumoto M Seya T. A distinct role of Riplet-mediated K63-Linked polyubiquitination of the RIG-I repressor domain in human antiviral innate immune responses. PLoS Pathog. (2013) 9:e1003533. 10.1371/journal.ppat.1003533 23950712 PMC3738492

[103] Cadena C Ahmad S Xavier A Willemsen J Park S Park JW Ubiquitin-dependent and -independent roles of E3 ligase RIPLET in innate immunity. Cell. (2019) 0:1187–200.e16. 10.1016/j.cell.2019.03.017 PMC6525047 31006531

[104] Gack MU Shin YC Joo C-H Urano T Liang C Sun L. TRIM25 RING-finger E3 ubiquitin ligase is essential for RIG-I-mediated antiviral activity. Nature. (2007) 446:916–20. 10.1038/nature05732 17392790

[105] Wang P Arjona A Zhang Y Sultana H Dai J Yang L. Caspase-12 controls West Nile virus infection via the viral RNA receptor RIG-I. Nat Immunol. (2010) 11:912–9. 10.1038/ni.1933 20818395 PMC3712356

[106] Lin H Jiang M Liu L Yang Z Ma Z Liu S. The long noncoding RNA Lnczc3h7a promotes a TRIM25-mediated RIG-I antiviral innate immune response. Nat Immunol. (2019) 20:812–23. 10.1038/s41590-019-0379-0 31036902

[107] Shi Y Yuan B Zhu W Zhang R Li L Hao X. Ube2D3 and Ube2N are essential for RIG-I-mediated MAVS aggregation in antiviral innate immunity. Nat Commun. (2017) 8:15138. 10.1038/ncomms15138 28469175 PMC5418627

[108] Liu Z Wu C Pan Y Liu H Wang X Yang Y. NDR2 promotes the antiviral immune response via facilitating TRIM25-mediated RIG-I activation in macrophages. Sci Adv. (2019) 5:eaav0163. 10.1126/sciadv.aav0163 30775439 PMC6365120

[109] Pauli E-K Chan YK Davis ME Gableske S Wang MK Feister KF. The ubiquitin-specific protease USP15 promotes RIG-I-mediated antiviral signaling by deubiquitylating TRIM25. Sci Signal. (2014) 7:ra3. 10.1126/scisignal.2004577 24399297 PMC4008495

[110] Liu HM Loo Y-M Horner SM Zornetzer GA Katze MG Gale M. The mitochondrial targeting chaperone 14-3-3ε regulates a RIG-I translocon that mediates membrane association and innate antiviral immunity. Cell Host Microbe. (2012) 11:528–37. 10.1016/j.chom.2012.04.006 22607805 PMC3358705

[111] Yan J Li Q Mao A-P Hu M-M Shu H-B. TRIM4 modulates type I interferon induction and cellular antiviral response by targeting RIG-I for K63-linked ubiquitination. J Mol Cell Biol. (2014) 6:154–63. 10.1093/jmcb/mju005 24755855

[112] Sanchez JG Chiang JJ Sparrer KMJ Alam SL Chi M Roganowicz MD. Mechanism of TRIM25 catalytic activation in the antiviral RIG-I pathway. Cell Rep. (2016) 16:1315–25. 10.1016/J.CELREP.2016.06.070 27425606 PMC5076470

[113] Gack MU Albrecht RA Urano T Inn K-S Huang I-C Carnero E. Influenza A virus NS1 targets the ubiquitin ligase TRIM25 to evade recognition by the host viral RNA sensor RIG-I. Cell Host Microbe. (2009) 5:439–49. 10.1016/j.chom.2009.04.006 19454348 PMC2737813

[114] Rajsbaum R Albrecht RA Wang MK Maharaj NP Versteeg GA Nistal-Villán E. Species-specific inhibition of RIG-I ubiquitination and IFN induction by the influenza A virus NS1 protein. PLoS Pathog. (2012) 8:e1003059. 10.1371/journal.ppat.1003059 23209422 PMC3510253

[115] Chiang C Pauli E-K Biryukov J Feister KF Meng M White EA. The human papillomavirus E6 oncoprotein targets USP15 and TRIM25 to suppress RIG-I-mediated innate immune signaling. J Virol. (2018) 92:e01737–17. 10.1128/JVI.01737-17 29263274 PMC5827370

[116] Koliopoulos MG Lethier M van der Veen AG Haubrich K Hennig J Kowalinski E. Molecular mechanism of influenza A NS1-mediated TRIM25 recognition and inhibition. Nat Commun. (2018) 9:1820. 10.1038/s41467-018-04214-8 29739942 PMC5940772

[117] Chen S-T Chen L Lin DS-C Chen S-Y Tsao Y-P Guo H. NLRP12 regulates anti-viral RIG-I activation via interaction with TRIM25. Cell Host Microbe. (2019) 25:602–16.e7. 10.1016/j.chom.2019.02.013 30902577 PMC6459718

[118] Zeng W Sun L Jiang X Chen X Hou F Adhikari A. Reconstitution of the RIG-I pathway reveals a signaling role of unanchored polyubiquitin chains in innate immunity. Cell. (2010) 141:315–30. 10.1016/j.cell.2010.03.029 20403326 PMC2919214

[119] Maelfait J Beyaert R. Emerging role of ubiquitination in antiviral RIG-I signaling. Microbiol Mol Biol Rev. (2012) 76:33–45. 10.1128/MMBR.05012-11 22390971 PMC3294425

[120] Sun X Xian H Tian S Sun T Qin Y Zhang S. A hierarchical mechanism of RIG-I ubiquitination provides sensitivity, robustness and synergy in antiviral immune responses. Sci Rep. (2016) 6:29263. 10.1038/srep29263 27387525 PMC4937349

[121] Xian H Xie W Yang S Liu Q Xia X Jin S. Stratified ubiquitination of RIG-I creates robust immune response and induces selective gene expression. Sci Adv. (2017) 3:e1701764. 10.1126/sciadv.1701764 28948228 PMC5609842

[122] Oshiumi H Matsumoto M Seya T. Ubiquitin-mediated modulation of the cytoplasmic viral RNA sensor RIG-I. J Biochem. (2012) 151:5–11. 10.1093/jb/mvr111 21890623

[123] Zhao C Jia M Song H Yu Z Wang W Li Q. The E3 ubiquitin ligase TRIM40 attenuates antiviral immune responses by targeting MDA5 and RIG-I. Cell Rep. (2017) 21:1613–23. 10.1016/J.CELREP.2017.10.020 29117565

[124] Okamoto M Kouwaki T Fukushima Y Oshiumi H. Regulation of RIG-I activation by K63-linked polyubiquitination. Front Immunol. (2017) 8:1942. 10.3389/fimmu.2017.01942 29354136 PMC5760545

[125] Lian H Zang R Wei J Ye W Hu M-M Chen Y-D. The zinc-finger protein ZCCHC3 binds RNA and facilitates viral RNA sensing and activation of the RIG-I-like receptors. Immunity. (2018) 49:438–48.e5. 10.1016/j.immuni.2018.08.014 30193849

[126] Zhang H-L Ye H-Q Liu S-Q Deng C-L Li X-D Shi P-Y. West Nile virus NS1 antagonizes interferon beta production by targeting RIG-I and MDA5. J Virol. (2017) 91:e02396–16. 10.1128/JVI.02396-16 28659477 PMC5571242

[127] Lang X Tang T Jin T Ding C Zhou R Jiang W. TRIM65-catalized ubiquitination is essential for MDA5-mediated antiviral innate immunity. J Exp Med. (2017) 214:459–73. 10.1084/jem.20160592 28031478 PMC5294850

[128] Peisley A Wu B Xu H Chen ZJ Hur S. Structural basis for ubiquitin-mediated antiviral signal activation by RIG-I. Nature. (2014) 509:110–4. 10.1038/NATURE13140 24590070 PMC6136653

[129] Tang ED Wang C-Y. MAVS self-association mediates antiviral innate immune signaling. J Virol. (2009) 83:3420–8. 10.1128/JVI.02623-08 19193783 PMC2663242

[130] Hou F Sun L Zheng H Skaug B Jiang QX Chen ZJ. MAVS forms functional prion-like aggregates to activate and propagate antiviral innate immune response. Cell. (2011) 146:448–61. 10.1016/j.cell.2011.06.041 21782231 PMC3179916

[131] Xu H He X Zheng H Huang LJ Hou F Yu Z. Structural basis for the prion-like MAVS filaments in antiviral innate immunity. Elife. (2014) 3:e1489. 10.7554/eLife.01489 24569476 PMC3932521

[132] He L Lührs T Ritter C. Solid-state NMR resonance assignments of the filament-forming CARD domain of the innate immunity signaling protein MAVS. Biomol NMR Assign. (2015) 9:223–7. 10.1007/s12104-014-9579-6 25301530

[133] Wu B Hur S. How RIG-I like receptors activate MAVS. Curr Opin Virol. (2015) 12:91–8. 10.1016/j.coviro.2015.04.004 25942693 PMC4470786

[134] Luo D Kohlway A Vela A Pyle AM. Visualizing the determinants of viral RNA recognition by innate immune sensor RIG-I. Structure. (2012) 20:1983–8. 10.1016/j.str.2012.08.029 23022350 PMC3515076

[135] Kohlway A Luo D Rawling DC Ding SC Pyle AM. Defining the functional determinants for RNA surveillance by RIG-I. EMBO Rep. (2013) 14:772–9. 10.1038/embor.2013.108 23897087 PMC3790051

[136] Linehan MM Dickey TH Molinari ES Fitzgerald ME Potapova O Iwasaki A. A minimal RNA ligand for potent RIG-I activation in living mice. Sci Adv. (2018) 4:e1701854. 10.1126/sciadv.1701854 29492454 PMC5821489

[137] Peisley A Lin C Wu B Orme-Johnson M Liu M Walz T. Cooperative assembly and dynamic disassembly of MDA5 filaments for viral dsRNA recognition. Proc Natl Acad Sci USA. (2011) 108:21010–5. 10.1073/pnas.1113651108 22160685 PMC3248507

[138] Berke IC Yu X Modis Y Egelman EH. MDA5 assembles into a polar helical filament on dsRNA. Proc Natl Acad Sci USA. (2012) 109:18437–41. 10.1073/pnas.1212186109 23090998 PMC3494895

[139] Lin J-P Fan Y-K Liu HM. The 14-3-3η chaperone protein promotes antiviral innate immunity via facilitating MDA5 oligomerization and intracellular redistribution. PLoS Pathog. (2019) 15:e1007582. 10.1371/journal.ppat.1007582 30742689 PMC6386420

[140] Zheng Y Gao C. E3 ubiquitin ligases, the powerful modulator of innate antiviral immunity. Cell Immunol. (2019) 340:103915. 10.1016/J.CELLIMM.2019.04.003 31054776

[141] Patel JR Jain A Chou Y Baum A Ha T García-Sastre A. ATPase-driven oligomerization of RIG-I on RNA allows optimal activation of type-I interferon. EMBO Rep. (2013) 14:780–7. 10.1038/embor.2013.102 23846310 PMC3790048

[142] Peisley A Wu B Yao H Walz T Hur S. RIG-I forms signaling-competent filaments in an ATP-dependent, ubiquitin-independent manner. Mol Cell. (2013) 51:573–83. 10.1016/J.MOLCEL.2013.07.024 23993742

[143] Binder M Eberle F Seitz S Mücke N Hüber CM Kiani N. Molecular mechanism of signal perception and integration by the innate immune sensor retinoic acid-inducible gene-I (RIG-I). J Biol Chem. (2011) 286:27278–87. 10.1074/jbc.M111.256974 21659521 PMC3149321

[144] Devarkar SC Schweibenz B Wang C Marcotrigiano J Patel SS. RIG-I uses an ATPase-powered translocation-throttling mechanism for kinetic proofreading of RNAs and oligomerization. Mol Cell. (2018) 72:355–68.e4. 10.1016/j.molcel.2018.08.021 30270105 PMC6434538

[145] Sohn J Hur S. Filament assemblies in foreign nucleic acid sensors. Curr Opin Struct Biol. (2016) 37:134–44. 10.1016/j.sbi.2016.01.011 26859869 PMC5070476

[146] Kawai T Takahashi K Sato S Coban C Kumar H Kato H. IPS-1, an adaptor triggering RIG-I- and Mda5-mediated type I interferon induction. Nat Immunol. (2005) 6:981–8. 10.1038/ni1243 16127453

[147] Xu L-G Wang Y-Y Han K-J Li L-Y Zhai Z Shu H-B. VISA is an adapter protein required for virus-triggered IFN-β signaling. Mol Cell. (2005) 19:727–40. 10.1016/J.MOLCEL.2005.08.014 16153868

[148] Seth RB Sun L Ea C-K Chen ZJ Identification and characterization of MAVS, a mitochondrial antiviral signaling protein that activates NF-κB and IRF3. Cell. (2005) 122:669–82. 10.1016/J.CELL.2005.08.012 16125763

[149] Hu J Nistal-Villán E Voho A Ganee A Kumar M Ding Y. A common polymorphism in the caspase recruitment domain of RIG-I modifies the innate immune response of human dendritic cells. J Immunol. (2010) 185:424–32. 10.4049/jimmunol.0903291 20511549 PMC2917324

[150] Marcus PI Sekellick MJ. Defective interfering particles with covalently linked [+/-]RNA induce interferon. Nature. (1977) 266:815–9. 10.1038/266815a0 194158

[151] Ferrage F Dutta K Nistal-Villán E Patel JR Sánchez-Aparicio MT De Ioannes P. Structure and dynamics of the second CARD of human RIG-I provide mechanistic insights into regulation of RIG-I activation. Structure. (2012) 20:2048–61. 10.1016/j.str.2012.09.003 23063562 PMC3625992

[152] Takeuchi O Akira S. Pattern recognition receptors and inflammation. Cell. (2010) 140:805–20. 10.1016/j.cell.2010.01.022 20303872

[153] Arnoult D Soares F Tattoli I Girardin SE. Mitochondria in innate immunity. EMBO Rep. (2011) 12:901–10. 10.1038/EMBOR.2011.157 21799518 PMC3166463

[154] Schröder M Baran M Bowie AG. Viral targeting of DEAD box protein 3 reveals its role in TBK1/IKKepsilon-mediated IRF activation. EMBO J. (2008) 27:2147–57. 10.1038/emboj.2008.143 18636090 PMC2516890

[155] Ng CT Sullivan BM Teijaro JR Lee AM Welch M Rice S Blockade of interferon beta, but not interferon alpha, signaling controls persistent viral infection. Cell Host Microbe. (2015) 17:653–61. 10.1016/j.chom.2015.04.005 25974304 PMC4432251

[156] Tsau JS Huang X Lai C-Y Hedrick SM. The effects of dendritic cell hypersensitivity on persistent viral infection. J Immunol. (2018) 200:1335–46. 10.4049/jimmunol.1601870 29311359 PMC5863746

[157] Camp JV Jonsson CB. A role for neutrophils in viral respiratory disease. Front Immunol. (2017) 8:550. 10.3389/fimmu.2017.00550 28553293 PMC5427094

[158] Gilliet M Cao W Liu Y-J. Plasmacytoid dendritic cells: sensing nucleic acids in viral infection and autoimmune diseases. Nat Rev Immunol. (2008) 8:594–606. 10.1038/nri2358 18641647

[159] Kong L Sun L Zhang H Liu Q Liu Y Qin L. An essential role for RIG-I in toll-like receptor-stimulated phagocytosis. Cell Host Microbe. (2009) 6:150–61. 10.1016/j.chom.2009.06.008 19683681

[160] Ma H Han P Ye W Chen H Zheng X Cheng L. The long noncoding RNA NEAT1 exerts antihantaviral effects by acting as positive feedback for RIG-I signaling. J Virol. (2017) 91:e02250–16. 10.1128/JVI.02250-16 28202761 PMC5391460

[161] Marie I Durbin JE Levy D. Differential viral induction of distinct interferon-alpha genes by positive feedback through interferon regulatory factor-7. EMBO J. (1998) 17:6660–9. 10.1093/emboj/17.22.6660 9822609 PMC1171011

[162] Haller O Kochs G Weber F. The interferon response circuit: Induction and suppression by pathogenic viruses. Virology. (2006) 344:119–30. 10.1016/j.virol.2005.09.024 16364743 PMC7125643

[163] Hui KPY Lee SMY Cheung CY Mao H Lai AKW Chan RWY. H5N1 influenza virus-induced mediators upregulate RIG-I in uninfected cells by paracrine effects contributing to amplified cytokine cascades. J Infect Dis. (2011) 204:1866–78. 10.1093/infdis/jir665 22013225

[164] Shaw AE Hughes J Gu Q Behdenna A Singer JB Dennis T. Fundamental properties of the mammalian innate immune system revealed by multispecies comparison of type I interferon responses. PLoS Biol. (2017) 15:e2004086. 10.1371/journal.pbio.2004086 29253856 PMC5747502

[165] Cui X-F Imaizumi T Yoshida H Borden EC Satoh K. Retinoic acid-inducible gene-I is induced by interferon-γ and regulates the expression of interferon-γ stimulated gene 15 in MCF-7 cells. Biochem Cell Biol. (2004) 82:401–5. 10.1139/o04-041 15181474

[166] Imaizumi T Yagihashi N Hatakeyama M Yamashita K Ishikawa A Taima K. Expression of retinoic acid-inducible gene-I in vascular smooth muscle cells stimulated with interferon-γ. Life Sci. (2004) 75:1171–80. 10.1016/J.LFS.2004.01.030 15219805

[167] Yount JS Moran TM López CB. Cytokine-independent upregulation of MDA5 in viral infection. J Virol. (2007) 81:7316–9. 10.1128/JVI.00545-07 17475649 PMC1933291

[168] Schmidt A Schwerd T Hamm W Hellmuth JC Cui S Wenzel M. 5′-triphosphate RNA requires base-paired structures to activate antiviral signaling via RIG-I. Proc Natl Acad Sci USA. (2009) 106:12067–72. 10.1073/pnas.0900971106 19574455 PMC2705279

[169] Marq J-B Kolakofsky D Garcin D Unpaired 5' ppp-nucleotides, as found in arenavirus double-stranded RNA panhandles, are not recognized by RIG-I. J Biol Chem. (2010) 285:18208–16. 10.1074/jbc.M109.089425 20400512 PMC2881745

[170] Schlee M Roth A Hornung V Hagmann CA Wimmenauer V Barchet W. Recognition of 5' triphosphate by RIG-I helicase requires short blunt double-stranded RNA as contained in panhandle of negative-strand virus. Immunity. (2009) 31:25–34. 10.1016/j.immuni.2009.05.008 19576794 PMC2824854

[171] Marq J-B Hausmann S Veillard N Kolakofsky D Garcin D. Short double-stranded RNAs with an overhanging 5' ppp-nucleotide, as found in arenavirus genomes, act as RIG-I decoys. J Biol Chem. (2011) 286:6108–16. 10.1074/jbc.M110.186262 21159780 PMC3057789

[172] Lu C Ranjith-Kumar CT Hao L Kao CC Li P. Crystal structure of RIG-I C-terminal domain bound to blunt-ended double-strand RNA without 5' triphosphate. Nucleic Acids Res. (2011) 39:1565–75. 10.1093/nar/gkq974 20961956 PMC3045611

[173] Goubau D Schlee M Deddouche S Pruijssers AJ Zillinger T Goldeck M. Antiviral immunity via RIG-I-mediated recognition of RNA bearing 5′-diphosphates. Nature. (2014) 514:372–5. 10.1038/nature13590 25119032 PMC4201573

[174] Kumar A Satpati P. Energetics of preferential binding of retinoic acid-inducible gene-I to double-stranded viral RNAs with 5′ Tri-/Diphosphate over 5′ Monophosphate. ACS Omega. (2018) 3:3786–95. 10.1021/acsomega.7b02019 30023880 PMC6044841

[175] Ren X Linehan MM Iwasaki A Pyle AM. RIG-I selectively discriminates against 5'-monophosphate RNA. Cell Rep. (2019) 26:2019–27.e4. 10.1016/j.celrep.2019.01.107 30784585

[176] Rawling DC Fitzgerald ME Pyle AM. Establishing the role of ATP for the function of the RIG-I innate immune sensor. Elife. (2015) 4:e09391. 10.7554/eLife.09391 26371557 PMC4622095

[177] Jiao X Chang JH Kilic T Tong L Kiledjian M. A mammalian pre-mRNA 5' end capping quality control mechanism and an unexpected link of capping to pre-mRNA processing. Mol Cell. (2013) 50:104–15. 10.1016/j.molcel.2013.02.017 23523372 PMC3630477

[178] Court DL Gan J Liang Y-H Shaw GX Tropea JE Costantino N. RNase III: genetics and function; structure and mechanism. Annu Rev Genet. (2013) 47:405–31. 10.1146/annurev-genet-110711-155618 24274754 PMC6311387

[179] Malathi K Saito T Crochet N Barton DJ Gale M Silverman RH. RNase L releases a small RNA from HCV RNA that refolds into a potent PAMP. RNA. (2010) 16:2108–19. 10.1261/rna.2244210 20833746 PMC2957051

[180] Wiatrek DM Candela ME Sedmík J Oppelt J Keegan LP O'Connell MA. Activation of innate immunity by mitochondrial dsRNA in mouse cells lacking p53 protein. RNA. (2019) 25:713–26. 10.1261/rna.069625.118 30894411 PMC6521600

[181] Chakrabarti A Jha BK Silverman RH. New insights into the role of RNase L in innate immunity. J Interferon Cytokine Res. (2011) 31:49–57. 10.1089/jir.2010.0120 21190483 PMC3021357

[182] Li X Lu C Stewart M Xu H Strong RK Igumenova T. Structural basis of double-stranded RNA recognition by the RIG-I like receptor MDA5. Arch Biochem Biophys. (2009) 488:23–33. 10.1016/J.ABB.2009.06.008 19531363

[183] Li X Ranjith-Kumar CT Brooks MT Dharmaiah S Herr AB Kao C. The RIG-I-like receptor LGP2 recognizes the termini of double-stranded RNA. J Biol Chem. (2009) 284:13881–91. 10.1074/jbc.M900818200 19278996 PMC2679488

[184] Sarkar D Desalle R Fisher PB Goodman M. Evolution of MDA-5/RIG-I-dependent innate immunity: independent evolution by domain grafting. Proc Natl Acad Sci USA. (2008) 105:17040–5. 10.1073/pnas.0804956105 18971330 PMC2579374

[185] Saito T Hirai R Loo Y-M Owen D Johnson CL Sinha SC. Regulation of innate antiviral defenses through a shared repressor domain in RIG-I and LGP2. Proc Natl Acad Sci USA. (2007) 104:582–7. 10.1073/pnas.0606699104 17190814 PMC1766428

[186] Luthra P Sun D Silverman RH He B Activation of IFN-β expression by a viral mRNA through RNase L and MDA5. Proc Natl Acad Sci USA. (2011) 108:2118–23. 10.1073/pnas.1012409108 21245317 PMC3033319

[187] Chow KT Gale M Loo Y-M. RIG-I and Other RNA Sensors in Antiviral Immunity. Annu Rev Immunol. (2018) 36:667–94. 10.1146/annurev-immunol-042617-053309 29677479

[188] Chiu Y-H Macmillan JB Chen ZJ. RNA polymerase III detects cytosolic DNA and induces type I interferons through the RIG-I pathway. Cell. (2009) 138:576–91. 10.1016/j.cell.2009.06.015 19631370 PMC2747301

[189] Ablasser A Bauernfeind F Hartmann G Latz E Fitzgerald KA Hornung V. RIG-I-dependent sensing of poly(dA:dT) through the induction of an RNA polymerase III–transcribed RNA intermediate. Nat Immunol. (2009) 10:1065–72. 10.1038/ni.1779 19609254 PMC3878616

[190] Chiang C Beljanski V Yin K Olagnier D Ben Yebdri F Steel C. Sequence-specific modifications enhance the broad-spectrum antiviral response activated by RIG-I agonists. J Virol. (2015) 89:8011–25. 10.1128/JVI.00845-15 26018150 PMC4505665

[191] Ho V Yong HY Chevrier M Narang V Lum J Toh Y-X. RIG-I activation by a designer short RNA ligand protects human immune cells against dengue virus infection without causing cytotoxicity. J Virol. (2019) 93:e00102–19. 10.1128/JVI.00102-19 31043531 PMC6600207

[192] Xu J Mercado-López X Grier JT Kim W Chun LF Irvine EB. Identification of a natural viral RNA motif that optimizes sensing of viral RNA by RIG-I. MBio. (2015) 6:e01265–15. 10.1128/mBio.01265-15 26443454 PMC4611036

[193] Runge S Sparrer KMJ Lässig C Hembach K Baum A García-Sastre A. *In vivo* ligands of MDA5 and RIG-I in measles virus-infected cells. PLoS Pathog. (2014) 10:e1004081. 10.1371/journal.ppat.1004081 24743923 PMC3990713

[194] Davis WG Bowzard JB Sharma SD Wiens ME Ranjan P Gangappa S. The 3' untranslated regions of influenza genomic sequences are 5'PPP-independent ligands for RIG-I. PLoS ONE. (2012) 7:e32661. 10.1371/journal.pone.0032661 22438882 PMC3305289

[195] Zhang Y Dittmer DP Mieczkowski PA Host KM Fusco WG Duncan JA. RIG-I detects Kaposi's sarcoma-associated herpesvirus transcripts in a RNA polymerase III-independent manner. MBio. (2018) 9:e00823–18. 10.1128/mBio.00823-18 29970461 PMC6030556

[196] Saito T Owen DM Jiang F Marcotrigiano J Gale M Jr. Innate immunity induced by composition-dependent RIG-I recognition of hepatitis C virus RNA. Nature. (2008) 454:523–7. 10.1038/nature07106 18548002 PMC2856441

[197] Uzri D Gehrke L. Nucleotide sequences and modifications that determine RIG-I/RNA binding and signaling activities. J Virol. (2009) 83:4174–84. 10.1128/JVI.02449-08 19224987 PMC2668486

[198] Jiang M Zhang S Yang Z Lin H Zhu J Liu L. Self-recognition of an inducible host lncRNA by RIG-I feedback restricts innate immune response. Cell. (2018) 173:906–19.e13. 10.1016/j.cell.2018.03.064 29706547

[199] Gebhardt A Laudenbach BT Pichlmair A. Discrimination of self and non-self ribonucleic acids. J Interf Cytokine Res. (2017) 37:184–97. 10.1089/jir.2016.0092 28475460 PMC5439445

[200] Huang AS Baltimore D. Defective viral particles and viral disease processes. Nature. (1970) 226:325–7. 10.1038/226325a0 5439728

[201] Huang AS. Defective interfering viruses. Annu Rev Microbiol. (1973) 27:101–18. 10.1146/annurev.mi.27.100173.000533 4356530

[202] Leppert M Kort L Kolakofsky D. Further characterization of Sendai virus DI-RNAs: a model for their generation. Cell. (1977) 12:539–52. 10.1016/0092-8674(77)90130-1 199355

[203] Nichol ST O'Hara PJ Holland JJ Perrault J. Structure and origin of a novel class of defective interfering particle of vesicular stomatitis virus. Nucleic Acids Res. (1984) 12:2775–90. 10.1093/nar/12.6.2775 6324126 PMC318705

[204] Strahle L Marq J-B Brini A Hausmann S Kolakofsky D Garcin D. Activation of the beta interferon promoter by unnatural sendai virus infection requires RIG-I and is inhibited by viral C proteins. J Virol. (2007) 81:12227–37. 10.1128/jvi.01300-07 17804509 PMC2169027

[205] Baum A Sachidanandam R García-Sastre A. Preference of RIG-I for short viral RNA molecules in infected cells revealed by next-generation sequencing. Proc Natl Acad Sci USA. (2010) 107:16303–8. 10.1073/pnas.1005077107 20805493 PMC2941304

[206] Baum A García-Sastre A. Differential recognition of viral RNA by RIG-I. Virulence. (2011) 2:166–9. 10.4161/viru.2.2.15481 21422808 PMC3100765

[207] Ho T-H Kew C Lui P-Y Chan C-P Satoh T Akira S. PACT- and RIG-I-dependent activation of type I interferon production by a defective interfering RNA derived from measles virus vaccine. J Virol. (2016) 90:1557–68. 10.1128/JVI.02161-15 26608320 PMC4719617

[208] Liu G Lu Y Liu Q Zhou Y. Inhibition of ongoing influenza A virus replication reveals different mechanisms of RIG-I activation. J Virol. (2019) 93:e02066–18. 10.1128/JVI.02066-18 30602605 PMC6401434

[209] Liu G Park H-S Pyo H-M Liu Q Zhou Y. Influenza A virus panhandle structure is directly involved in RIG-I activation and interferon induction. J Virol. (2015) 89:6067–79. 10.1128/JVI.00232-15 25810557 PMC4442436

[210] Nayak DP Sivasubramanian N The structure of influenza virus defective interfering (DI) RNAs and their progenitor genes. In: Palese P Kingsbury DW editors. Genetics of Influenza Viruses. Vienna: Springer Vienna (1983). p. 255–79. 10.1007/978-3-7091-8706-7_8

[211] Yount JS Gitlin L Moran TM López CB. MDA5 participates in the detection of paramyxovirus infection and is essential for the early activation of dendritic cells in response to sendai virus defective interfering particles. J Immunol. (2008) 180:4910–8. 10.4049/JIMMUNOL.180.7.4910 18354215

[212] Yount JS Kraus TA Horvath CM Moran TM López CB. A novel role for viral-defective interfering particles in enhancing dendritic cell maturation. J Immunol. (2006) 177:4503–13. 10.4049/JIMMUNOL.177.7.4503 16982887

[213] Dimmock NJ Easton AJ. Defective interfering influenza virus RNAs: time to reevaluate their clinical potential as broad-spectrum antivirals? J Virol. (2014) 88:5217–27. 10.1128/JVI.03193-13 24574404 PMC4019098

[214] Weinberger B Herndler-Brandstetter D Schwanninger A Weiskopf D Grubeck-Loebenstein B. Biology of immune responses to vaccines in elderly persons. Clin Infect Dis. (2008) 46:1078–84. 10.1086/529197 18444828

[215] Chen WH Kozlovsky BF Effros RB Grubeck-Loebenstein B Edelman R Sztein MB. Vaccination in the elderly: an immunological perspective. Trends Immunol. (2009) 30:351–9. 10.1016/j.it.2009.05.002 19540808 PMC3739436

[216] Derhovanessian E Pawelec G. Vaccination in the elderly. Microb Biotechnol. (2012) 5:226–32. 10.1111/j.1751-7915.2011.00283.x 21880118 PMC3815782

[217] Ciabattini A Nardini C Santoro F Garagnani P Medaglini D. Vaccination in the elderly: the challenge of immune changes with aging. Semin Immunol. (2018) 40:83–94. 10.1016/J.SMIM.2018.10.010 30501873

[218] Molony RD Nguyen JT Kong Y Montgomery RR Shaw AC Iwasaki A. Aging impairs both primary and secondary RIG-I signaling for interferon induction in human monocytes. Sci Signal. (2017) 10:eaan2392. 10.1126/scisignal.aan2392 29233916 PMC6429941

[219] Newall AT Chen C Wood JG Stockwell MS. Within-season influenza vaccine waning suggests potential net benefits to delayed vaccination in older adults in the United States. Vaccine. (2018) 36:5910–5. 10.1016/J.VACCINE.2018.08.007 30146403

[220] Oldstone MB. Viral persistence: mechanisms and consequences. Curr Opin Microbiol. (1998) 1:436–41. 10.1016/S1369-5274(98)80062-3 10066504

[221] Manzoni TB López CB. Defective (interfering) viral genomes re-explored: impact on antiviral immunity and virus persistence. Future Virol. (2018) 13:493–503. 10.2217/fvl-2018-0021 30245734 PMC6136085

[222] Stauffer Thompson KA Rempala GA Yin J. Multiple-hit inhibition of infection by defective interfering particles. J Gen Virol. (2009) 90:888–99. 10.1099/vir.0.005249-0 19264636 PMC2889439

[223] Ziegler CM Eisenhauer P Bruce EA Beganovic V King BR Weir ME. A novel phosphoserine motif in the LCMV matrix protein Z regulates the release of infectious virus and defective interfering particles. J Gen Virol. (2016) 97:2084–9. 10.1099/jgv.0.000550 27421645 PMC5756487

[224] Ziegler C Eisenhauer P Manuelyan I Weir M Bruce E Ballif B. Host-driven phosphorylation appears to regulate the budding activity of the lassa virus matrix protein. Pathogens. (2018) 7:97. 10.3390/pathogens7040097 30544850 PMC6313517

[225] Rehwinkel J Reis e Sousa C. RIGorous detection: exposing virus through RNA sensing. Science. (2010) 327:284–6. 10.1126/science.1185068 20075242

[226] Rice GI Del Toro Duany Y Jenkinson EM Forte GM Anderson BH Ariaudo G. Gain-of-function mutations in IFIH1 cause a spectrum of human disease phenotypes associated with upregulated type I interferon signaling. Nat Genet. (2014) 46:503–9. 10.1038/ng.2933 24686847 PMC4004585

[227] Ahmad S Mu X Yang F Greenwald E Park JW Jacob E. Breaching self-tolerance to Alu duplex RNA underlies MDA5-mediated inflammation. Cell. (2018) 172:797. 10.1016/J.CELL.2017.12.016 29395326 PMC5807104

[228] Oda H Nakagawa K Abe J Awaya T Funabiki M Hijikata A. Aicardi-Goutières syndrome is caused by IFIH1 mutations. Am J Hum Genet. (2014) 95:121–5. 10.1016/j.ajhg.2014.06.007 24995871 PMC4085581

[229] Funabiki M Kato H Miyachi Y Toki H Motegi H Inoue M. Autoimmune disorders associated with gain of function of the intracellular sensor MDA5. Immunity. (2014) 40:199–212. 10.1016/J.IMMUNI.2013.12.014 24530055

[230] Schuberth-Wagner C Ludwig J Bruder AK Herzner A-M Zillinger T Goldeck M. A conserved histidine in the RNA sensor RIG-I controls immune tolerance to N1-2′O-methylated Self RNA. Immunity. (2015) 43:41–51. 10.1016/j.immuni.2015.06.015 26187414 PMC7128463

[231] Zheng J Yong HY Panutdaporn N Liu C Tang K Luo D. High-resolution HDX-MS reveals distinct mechanisms of RNA recognition and activation by RIG-I and MDA5. Nucleic Acids Res. (2015) 43:1216–30. 10.1093/nar/gku1329 25539915 PMC4333383

[232] Zheng J Wang C Chang MR Devarkar SC Schweibenz B Crynen GC. HDX-MS reveals dysregulated checkpoints that compromise discrimination against self RNA during RIG-I mediated autoimmunity. Nat Commun. (2018) 9:5366. 10.1038/s41467-018-07780-z 30560918 PMC6299088

[233] Lässig C Matheisl S Sparrer KMJM de Oliveira Mann CC Moldt M Patel JR. ATP hydrolysis by the viral RNA sensor RIG-I prevents unintentional recognition of self-RNA. Elife. (2015) 4:e10859. 10.7554/eLife.10859 26609812 PMC4733034

[234] Nallagatla SR Toroney R Bevilacqua PC. A brilliant disguise for self RNA: 5'-end and internal modifications of primary transcripts suppress elements of innate immunity. RNA Biol. (2008) 5:140–4. 10.4161/rna.5.3.6839 18769134 PMC2809118

[235] Gokhale NS Horner SM. RNA modifications go viral. PLoS Pathog. (2017) 13:e1006188. 10.1371/journal.ppat.1006188 28278189 PMC5344520

[236] Durbin AF Wang C Marcotrigiano J Gehrke L. RNAs containing modified nucleotides fail to trigger RIG-I conformational changes for innate immune signaling. MBio. (2016) 7:e00833–16. 10.1128/mBio.00833-16 27651356 PMC5030355

[237] Oshiumi H Mifsud EJ Daito T. Links between recognition and degradation of cytoplasmic viral RNA in innate immune response. Rev Med Virol. (2016) 26:90–101. 10.1002/rmv.1865 26643446

[238] Satoh T Kato H Kumagai Y Yoneyama M Sato S Matsushita K. LGP2 is a positive regulator of RIG-I- and MDA5-mediated antiviral responses. Proc Natl Acad Sci USA. (2010) 107:1512–7. 10.1073/pnas.0912986107 20080593 PMC2824407

[239] Civril F Bennett M Moldt M Deimling T Witte G Schiesser S. The RIG-I ATPase domain structure reveals insights into ATP-dependent antiviral signalling. EMBO Rep. (2011) 12:1127–34. 10.1038/embor.2011.190 21979817 PMC3207106

[240] Wilson RC Doudna JA. Molecular mechanisms of RNA interference. Annu Rev Biophys. (2013) 42:217–39. 10.1146/annurev-biophys-083012-130404 23654304 PMC5895182

[241] Peach SE York K Hesselberth JR. Global analysis of RNA cleavage by 5'-hydroxyl RNA sequencing. Nucleic Acids Res. (2015) 43:e108. 10.1093/nar/gkv536 26001965 PMC4787814

[242] Louber J Brunel J Uchikawa E Cusack S Gerlier D. Kinetic discrimination of self/non-self RNA by the ATPase activity of RIG-I and MDA5. BMC Biol. (2015) 13:54. 10.1186/s12915-015-0166-9 26215161 PMC4517655

[243] Yu Q Qu K Modis Y. Cryo-EM structures of MDA5-dsRNA filaments at different stages of ATP hydrolysis. Mol Cell. (2018) 72:999–1012.e6. 10.1016/J.MOLCEL.2018.10.012 30449722 PMC6310684

[244] Wang M Yu F Wu W Zhang Y Chang W Ponnusamy M. Circular RNAs: a novel type of non-coding RNA and their potential implications in antiviral immunity. Int J Biol Sci. (2017) 13:1497–506. 10.7150/ijbs.22531 29230098 PMC5723916

[245] Chen YG Kim MV Chen X Batista PJ Aoyama S Wilusz JE. Sensing self and foreign circular RNAs by intron identity. Mol Cell. (2017) 67:228–38.e5. 10.1016/j.molcel.2017.05.022 28625551 PMC5610545

[246] Eckard SC Rice GI Fabre A Badens C Gray EE Hartley JL. The SKIV2L RNA exosome limits activation of the RIG-I-like receptors. Nat Immunol. (2014) 15:839–45. 10.1038/ni.2948 25064072 PMC4139417

[247] Murai K Honda M Shirasaki T Shimakami T Omura H Misu H. Induction of selenoprotein P mRNA during hepatitis C virus infection inhibits RIG-I-mediated antiviral immunity. Cell Host Microbe. (2019) 25:588–601.e7. 10.1016/j.chom.2019.02.015 30974086

[248] Fan J Cheng M Chi X Liu X Yang W. Human long non-coding RNA LncATV promotes viral replication by restricting RIG-I-mediated innate immunity. Front. Immunol. (2019) 10:1711 10.3389/fimmu.2019.01711 PMC6658999 31379885

[249] Kim D Kim H Han S Scatena M Kim D-H Lee JB. Immunostimulatory effects triggered by self-assembled microspheres with tandem repeats of polymerized RNA strands. Adv Healthc Mater. (2019) 8:1801395. 10.1002/adhm.201801395 30657652

[250] Zhao K Du J Peng Y Li P Wang S Wang Y. LINE1 contributes to autoimmunity through both RIG-I- and MDA5-mediated RNA sensing pathways. J Autoimmun. (2018) 90:105–15. 10.1016/J.JAUT.2018.02.007 29525183

[251] Chiang JJ Sparrer KMJ van Gent M Lässig C Huang T Osterrieder N. Viral unmasking of cellular 5S rRNA pseudogene transcripts induces RIG-I-mediated immunity. Nat Immunol. (2018) 19:53–62. 10.1038/s41590-017-0005-y 29180807 PMC5815369

[252] Khan M Syed GH Kim S-J Siddiqui A. Mitochondrial dynamics and viral infections: a close nexus. Biochim Biophys Acta. (2015) 1853:2822–33. 10.1016/j.bbamcr.2014.12.040 25595529 PMC4500740

[253] Dhir A Dhir S Borowski LS Jimenez L Teitell M Rötig A. Mitochondrial double-stranded RNA triggers antiviral signalling in humans. Nature. (2018) 560:238–42. 10.1038/s41586-018-0363-0 30046113 PMC6570621

[254] Hardy M-P Audemard É Migneault F Feghaly A Brochu S Gendron P. Apoptotic endothelial cells release small extracellular vesicles loaded with immunostimulatory viral-like RNAs. Sci Rep. (2019) 9:7203. 10.1038/s41598-019-43591-y 31076589 PMC6510763

[255] Hoffmann H-H Schneider WM Rice CM. Interferons and viruses: an evolutionary arms race of molecular interactions. Trends Immunol. (2015) 36:124–38. 10.1016/j.it.2015.01.004 25704559 PMC4384471

[256] Schulz KS Mossman KL. Viral evasion strategies in type I IFN signaling - a summary of recent developments. Front Immunol. (2016) 7:498. 10.3389/fimmu.2016.00498 27891131 PMC5104748

[257] Chan YK Gack MU. Viral evasion of intracellular DNA and RNA sensing. Nat Rev Microbiol. (2016) 14:360–73. 10.1038/nrmicro.2016.45 27174148 PMC5072394

[258] García-Sastre A. Ten strategies of interferon evasion by viruses. Cell Host Microbe. (2017) 22:176–84. 10.1016/j.chom.2017.07.012 28799903 PMC5576560

[259] Weber M Weber F. Segmented negative-strand RNA viruses and RIG-I: divide (your genome) and rule. Curr Opin Microbiol. (2014) 20:96–102. 10.1016/J.MIB.2014.05.002 24930021

[260] Samji T. Influenza A: understanding the viral life cycle. Yale J Biol Med. (2009) 82:153–9. 20027280 PMC2794490

[261] Liu G Lu Y Thulasi Raman SN Xu F Wu Q Li Z. Nuclear-resident RIG-I senses viral replication inducing antiviral immunity. Nat Commun. (2018) 9:3199. 10.1038/s41467-018-05745-w 30097581 PMC6086882

[262] Habjan M Andersson I Klingström J Schümann M Martin A Zimmermann P. Processing of genome 5′ termini as a strategy of negative-strand RNA viruses to avoid RIG-I-dependent interferon induction. PLoS ONE. (2008) 3:e2032. 10.1371/journal.pone.0002032 18446221 PMC2323571

[263] Ingle H Kumar S Raut AA Mishra A Kulkarni DD Kameyama T. The microRNA miR-485 targets host and influenza virus transcripts to regulate antiviral immunity and restrict viral replication. Sci Signal. (2015) 8:ra126. 10.1126/scisignal.aab3183 26645583

[264] Yoshida A Kawabata R Honda T Sakai K Ami Y Sakaguchi T. A single amino acid substitution within the paramyxovirus sendai virus nucleoprotein is a critical determinant for production of interferon-beta-inducing copyback-type defective interfering genomes. J Virol. (2018) 92:e02094–17. 10.1128/JVI.02094-17 29237838 PMC5809723

[265] Te Velthuis AJW Long JC Bauer DLV Fan RLY Yen H-L Sharps J. Mini viral RNAs act as innate immune agonists during influenza virus infection. Nat Microbiol. (2018) 3:1234–42. 10.1038/s41564-018-0240-5 30224800 PMC6203953

[266] Nikonov A Mölder T Sikut R Kiiver K Männik A Toots U. RIG-I and MDA-5 detection of viral RNA-dependent RNA polymerase activity restricts positive-strand RNA virus replication. PLoS Pathog. (2013) 9:e1003610. 10.1371/journal.ppat.1003610 24039580 PMC3764220

[267] King BR Samacoits A Eisenhauer PL Ziegler CM Bruce EA Zenklusen D Visualization of arenavirus RNA species in individual cells by single-molecule fluorescence *in situ* hybridization (smFISH) suggests a model of cyclical infection and clearance during persistence. J Virol. (2018) 92:e02241–17. 10.1128/JVI.02241-17 29643234 PMC5974494

[268] Hastie KM Kimberlin CR Zandonatti MA MacRae IJ Saphire EO. Structure of the Lassa virus nucleoprotein reveals a dsRNA-specific 3' to 5' exonuclease activity essential for immune suppression. Proc Natl Acad Sci USA. (2011) 108:2396–401. 10.1073/pnas.1016404108 21262835 PMC3038715

[269] Huang Q Shao J Lan S Zhou Y Xing J Dong C. *In vitro* and *in vivo* characterizations of pichinde viral nucleoprotein exoribonuclease functions. J Virol. (2015) 89:6595–607. 10.1128/JVI.00009-15 25878103 PMC4468471

[270] Ma Y Wu L Shaw N Gao Y Wang J Sun Y. Structural basis and functional analysis of the SARS coronavirus nsp14-nsp10 complex. Proc Natl Acad Sci USA. (2015) 112:9436–41. 10.1073/pnas.1508686112 26159422 PMC4522806

[271] Durzynska I Sauerwald M Karl N Madhugiri R Ziebuhr J. Characterization of a bafinivirus exoribonuclease activity. J Gen Virol. (2018) 99:1253–60. 10.1099/jgv.0.001120 30058998

[272] Chiang JJ Davis ME Gack MU. Regulation of RIG-I-like receptor signaling by host and viral proteins. Cytokine Growth Factor Rev. (2014) 25:491–505. 10.1016/J.CYTOGFR.2014.06.005 25023063 PMC7108356

[273] Ling Z Tran KC Teng MN Human respiratory syncytial virus non-structural protein NS2 antagonizes the activation of beta interferon transcription by interacting with RIG-I. J Virol. (2009) 83:3734–42. 10.1128/JVI.02434-08 19193793 PMC2663251

[274] Xing J Ly H Liang Y The Z proteins of pathogenic but not non-pathogenic arenaviruses inhibit RIG-i-like receptor-dependent interferon production. J Virol. (2015) 89:2944–55. 10.1128/JVI.03349-14 25552708 PMC4325705

[275] Zhao J Zeng Y Xu S Chen J Shen G Yu C. A viral deamidase targets the helicase domain of RIG-I to block RNA-induced activation. Cell Host Microbe. (2016) 20:770–84. 10.1016/j.chom.2016.10.011 27866900 PMC5159239

[276] Li W Chen H Sutton T Obadan A Perez DR. Interactions between the Influenza A virus RNA polymerase components and retinoic acid-inducible gene I. J Virol. (2014) 88:10432–47. 10.1128/JVI.01383-14 24942585 PMC4178842

[277] Reynard S Russier M Fizet A Carnec X Baize S. Exonuclease domain of the Lassa virus nucleoprotein is critical to avoid RIG-I signaling and to inhibit the innate immune response. J Virol. (2014) 88:13923–7. 10.1128/JVI.01923-14 25253344 PMC4248989

[278] Shao J Huang Q Liu X Di D Liang Y Ly H Arenaviral nucleoproteins suppress PACT-induced augmentation of RIG-I function to inhibit type I interferon production. J Virol. (2018) 5:ofy277 10.1128/JVI.00482-18 PMC6002705 29669840

[279] Qin L Ren L Zhou Z Lei X Chen L Xue Q Rotavirus non-structural protein 1 antagonizes innate immune response by interacting with retinoic acid inducible gene I. Virol J. (2011) 8:526 10.1186/1743-422X-8-526 22152002 PMC3254192

[280] Motz C Schuhmann KM Kirchhofer A Moldt M Witte G Conzelmann K-K. Paramyxovirus V proteins disrupt the fold of the RNA sensor MDA5 to inhibit antiviral signaling. Science. (2013) 339:690–3. 10.1126/science.1230949 23328395

[281] Sánchez-Aparicio MT Feinman LJ García-Sastre A Shaw ML. Paramyxovirus V proteins interact with the RIG-I/TRIM25 regulatory complex and inhibit RIG-I signaling. J Virol. (2018) 92:e01960–17. 10.1128/JVI.01960-17 29321315 PMC5827389

[282] Xing J Wang S Lin R Mossman KL Zheng C. Herpes simplex virus 1 tegument protein US11 downmodulates the RLR signaling pathway via direct interaction with RIG-I and MDA-5. J Virol. (2012) 86:3528–40. 10.1128/JVI.06713-11 22301138 PMC3302539

[283] Liu Y Olagnier D Lin R. Host and viral modulation of RIG-I-mediated antiviral immunity. Front Immunol. (2017) 7:662. 10.3389/fimmu.2016.00662 28096803 PMC5206486

[284] Cui J Song Y Li Y Zhu Q Tan P Qin Y. USP3 inhibits type I interferon signaling by deubiquitinating RIG-I-like receptors. Cell Res. (2014) 24:400–16. 10.1038/cr.2013.170 24366338 PMC3975496

[285] Li H Zhao Z Ling J Pan L Zhao X Zhu H. USP14 promotes K63-linked RIG-I deubiquitination and suppresses antiviral immune responses. Eur J Immunol. (2018) 49:42–53. 10.1002/eji.201847603 30466171

[286] Jiang J Tang H. Mechanism of inhibiting type I interferon induction by hepatitis B virus X protein. Protein Cell. (2010) 1:1106–17. 10.1007/s13238-010-0141-8 21213104 PMC4875076

[287] Likai J Shasha L Wenxian Z Jingjiao M Jianhe S Hengan W. Porcine deltacoronavirus nucleocapsid protein suppressed IFN-β production by interfering porcine RIG-I dsRNA-binding and K63-linked polyubiquitination. Front Immunol. (2019) 10:1024. 10.3389/fimmu.2019.01024 31143181 PMC6521028

[288] Nguyen NTH Now H Kim W-J Kim N Yoo J-Y. Ubiquitin-like modifier FAT10 attenuates RIG-I mediated antiviral signaling by segregating activated RIG-I from its signaling platform. Sci Rep. (2016) 6:23377. 10.1038/srep23377 26996158 PMC4800306

[289] Jiang J Li J Fan W Zheng W Yu M Chen C. Robust Lys63-linked ubiquitination of RIG-I promotes cytokine eruption in early influenza B virus infection. J Virol. (2016) 90:6263–75. 10.1128/JVI.00549-16 27122586 PMC4936144

[290] Zhu J Zhang Y Ghosh A Cuevas RA Forero A Dhar J. Antiviral activity of human OASL protein is mediated by enhancing signaling of the RIG-I RNA sensor. Immunity. (2014) 40:936–48. 10.1016/j.immuni.2014.05.007 24931123 PMC4101812

[291] Arimoto K Takahashi H Hishiki T Konishi H Fujita T Shimotohno K. Negative regulation of the RIG-I signaling by the ubiquitin ligase RNF125. Proc Natl Acad Sci USA. (2007) 104:7500–5. 10.1073/PNAS.0611551104 17460044 PMC1863485

[292] Chen W Han C Xie B Hu X Yu Q Shi L. Induction of Siglec-G by RNA viruses inhibits the innate immune response by promoting RIG-I degradation. Cell. (2013) 152:467–78. 10.1016/j.cell.2013.01.011 23374343

[293] Zhao K Zhang Q Li X Zhao D Liu Y Shen Q Cytoplasmic STAT4 promotes antiviral type I IFN production by blocking CHIP-mediated degradation of RIG-I kai. J Immunol. (2016) 196:1209–17. 10.4049/jimmunol.1501224 26695369

[294] Du Y Duan T Feng Y Liu Q Lin M Cui J. LRRC25 inhibits type I IFN signaling by targeting ISG15-associated RIG-I for autophagic degradation. EMBO J. (2018) 37:351–66. 10.15252/embj.201796781 29288164 PMC5793803

[295] Xian H Yang S Jin S Zhang Y Cui J. LRRC59 modulates type I interferon signaling by restraining the SQSTM1/p62-mediated autophagic degradation of pattern recognition receptor DDX58/RIG-I. Autophagy. (2019). 10.1080/15548627.2019.1615303. [Epub ahead of print]. 31068071 PMC6999607

[296] Xia M Gonzalez P Li C Meng G Jiang A Wang H. Mitophagy enhances oncolytic measles virus replication by mitigating DDX58/RIG-I-like receptor signaling. J Virol. (2014) 88:5152–64. 10.1128/JVI.03851-13 24574393 PMC3993837

[297] Barral PM Sarkar D Fisher PB Racaniello VR. RIG-I is cleaved during picornavirus infection. Virology. (2009) 391:171–6. 10.1016/J.VIROL.2009.06.045 19628239 PMC2743091

[298] Zhu Z Wang G Yang F Cao W Mao R Du X. Foot-and-mouth disease virus viroporin 2B antagonizes RIG-I-mediated antiviral effects by inhibition of its protein expression. J Virol. (2016) 90:11106–21. 10.1128/JVI.01310-16 27707918 PMC5126369

[299] Barral PM Morrison JM Drahos J Gupta P Sarkar D Fisher PB. MDA-5 is cleaved in poliovirus-infected cells. J Virol. (2007) 81:3677–84. 10.1128/JVI.01360-06 17267501 PMC1866155

[300] Wang W Jiang M Liu S Zhang S Liu W Ma Y. RNF122 suppresses antiviral type I interferon production by targeting RIG-I CARDs to mediate RIG-I degradation. Proc Natl Acad Sci USA. (2016) 113:9581–6. 10.1073/pnas.1604277113 27506794 PMC5003265

[301] Zhou P Ding X Wan X Liu L Yuan X Zhang W. MLL5 suppresses antiviral innate immune response by facilitating STUB1-mediated RIG-I degradation. Nat Commun. (2018) 9:1243. 10.1038/s41467-018-03563-8 29593341 PMC5871759

[302] Matsumiya T Imaizumi T Yoshida H Satoh K Topham MK Stafforini DM. The levels of retinoic acid-inducible gene I are regulated by heat shock protein 90-alpha. J Immunol. (2009) 182:2717–25. 10.4049/jimmunol.0802933 19234166 PMC2722243

[303] Iwamura T Yoneyama M Koizumi N Okabe Y Namiki H Samuel CE. PACT, a double-stranded RNA binding protein acts as a positive regulator for type I interferon gene induced by Newcastle disease virus. Biochem Biophys Res Commun. (2001) 282:515–23. 10.1006/BBRC.2001.4606 11401490

[304] Kok K-HH Lui P-YY Ng M-HJHJ Siu K-LL Au SWN Jin D-YY. The double-stranded RNA-binding protein PACT functions as a cellular activator of RIG-I to facilitate innate antiviral response. Cell Host Microbe. (2011) 9:299–309. 10.1016/J.CHOM.2011.03.007 21501829

[305] Chen K-R Chang C-H Huang C-Y Lin C-Y Lin W-Y Lo Y-C. TBK1-associated protein in endolysosomes (TAPE)/CC2D1A is a key regulator linking RIG-I-like receptors to antiviral immunity. J Biol Chem. (2012) 287:32216–21. 10.1074/jbc.C112.394346 22833682 PMC3442552

[306] Suzuki T Oshiumi H Miyashita M Aly HH Matsumoto M Seya T. Cell type-specific subcellular localization of phospho-TBK1 in response to cytoplasmic viral DNA. PLoS ONE. (2013) 8:e83639. 10.1371/journal.pone.0083639 24349538 PMC3857317

[307] Onorati M Li Z Liu F Sousa AMM Nakagawa N Li M. Zika virus disrupts phospho-TBK1 localization and mitosis in human neuroepithelial stem cells and radial glia. Cell Rep. (2016) 16:2576–92. 10.1016/j.celrep.2016.08.038 27568284 PMC5135012

[308] Li S Lu L-F Li Z-C Zhang C Zhou X-Y Zhou Y. Zebrafish MVP recruits and degrades TBK1 to suppress IFN production. J Immunol. (2018) 202:559–66. 10.4049/jimmunol.1801325 30530482

[309] Outlioua A Pourcelot M Arnoult D. The role of optineurin in antiviral type I interferon production. Front Immunol. (2018) 9:853. 10.3389/fimmu.2018.00853 29755463 PMC5932347

[310] Ibsen MS Gad HH Andersen LL Hornung V Julkunen I Sarkar SN. Structural and functional analysis reveals that human OASL binds dsRNA to enhance RIG-I signaling. Nucleic Acids Res. (2015) 43:5236–48. 10.1093/nar/gkv389 25925578 PMC4446440

[311] Beattie E Denzler KL Tartaglia J Perkus ME Paoletti E Jacobs BL. Reversal of the interferon-sensitive phenotype of a vaccinia virus lacking E3L by expression of the reovirus S4 gene. J Virol. (1995) 69:499–505. 7527085 10.1128/jvi.69.1.499-505.1995 PMC188598

[312] Kok KH Ng M-HJ Ching Y-P Jin D-Y. Human TRBP and PACT directly interact with each other and associate with dicer to facilitate the production of small interfering RNA. J Biol Chem. (2007) 282:17649–57. 10.1074/jbc.M611768200 17452327

[313] Schlee M Hartmann E Coch C Wimmenauer V Janke M Barchet W. Approaching the RNA ligand for RIG-I? Immunol Rev. (2009) 227:66–74. 10.1111/j.1600-065X.2008.00724.x 19120476

[314] Brisse M Ly H Brisse M Ly H Viral inhibitions of PACT-induced RIG-I activation. Oncotarget. (2017) 5: 60725–6. 10.18632/oncotarget.18928 PMC5617381 28977821

[315] Brisse ME Ly H. Hemorrhagic fever-causing arenaviruses: lethal pathogens and potent immune suppressors. Front Immunol. (2019) 10:372. 10.3389/fimmu.2019.00372 30918506 PMC6424867

[316] Chen J Fang P Wang M Peng Q Ren J Wang D. Porcine deltacoronavirus nucleocapsid protein antagonizes IFN-β production by impairing dsRNA and PACT binding to RIG-I. Virus Genes. (2019). 10.1007/s11262-019-01673-z. [Epub ahead of print]. 31129785 PMC7088841

[317] Chen H Li Y Zhang J Ran Y Wei J Yang Y. RAVER1 is a coactivator of MDA5-mediated cellular antiviral response. J Mol Cell Biol. (2013) 5:111–9. 10.1093/jmcb/mjt006 23390309

[318] Yang Q Bai S-Y Li L-F Li S Zhang Y Munir M. Human hemoglobin subunit beta functions as a pleiotropic regulator of the RIG-I/MDA5-mediated antiviral innate immune responses. J Virol. (2019). 10.1128/JVI.00718-19. [Epub ahead of print]. 31167908 PMC6675906

[319] Patel RC Sen GC. PACT, a protein activator of the interferon-induced protein kinase, PKR. EMBO J. (1998) 17:4379–90. 10.1093/emboj/17.15.4379 9687506 PMC1170771

[320] Marques JT White CL Peters GA Williams BRG Sen GC. The role of PACT in mediating gene induction, PKR activation, and apoptosis in response to diverse stimuli. J Interferon Cytokine Res. (2008) 28:469–76. 10.1089/jir.2007.0006 18729737 PMC2965581

[321] Yoo J-S Takahasi K Ng CS Ouda R Onomoto K Yoneyama M. DHX36 enhances RIG-I signaling by facilitating PKR-mediated antiviral stress granule formation. PLoS Pathog. (2014) 10:e1004012. 10.1371/journal.ppat.1004012 24651521 PMC3961341

[322] Yoneyama M Jogi M Onomoto K. Regulation of antiviral innate immune signaling by stress-induced RNA granules. J Biochem. (2016) 159:mvv122. 10.1093/jb/mvv122 26748340 PMC4763080

[323] Onomoto K Jogi M Yoo J-S Narita R Morimoto S Takemura A. Critical role of an antiviral stress granule containing RIG-I and PKR in viral detection and innate immunity. PLoS ONE. (2012) 7:e43031. 10.1371/journal.pone.0043031 22912779 PMC3418241

[324] Sánchez-Aparicio MT Ayllón J Leo-Macias A Wolff T García-Sastre A. Subcellular localizations of RIG-I, TRIM25, and MAVS complexes. J Virol. (2017) 91:e01155–16. 10.1128/JVI.01155-16 27807226 PMC5215348

[325] Pham AM Santa Maria FG Lahiri T Friedman E Marié IJ Levy DE. PKR transduces MDA5-dependent signals for type I IFN induction. PLoS Pathog. (2016) 12:e1005489. 10.1371/journal.ppat.1005489 26939124 PMC4777437

[326] Li L-F Yu J Zhang Y Yang Q Li Y Zhang L. Interferon-inducible oligoadenylate synthetase-like protein acts as an antiviral effector against classical swine fever virus via the MDA5-mediated type I interferon-signaling pathway. J Virol. (2017) 91:e01514–16. 10.1128/JVI.01514-16 28331099 PMC5432864

[327] Xu L Xiao N Liu F Ren H Gu J Beutler BA. Inhibition of RIG-I and MDA5-dependent antiviral response by gC1qR at mitochondria. PNAS. (2009) 106:1530–5. 10.1073/pnas.0811029106 19164550 PMC2635802

[328] Ling T Li S-N Weng G-X Wang W Li C Cao L. TARBP2 negatively regulates IFN-β production and innate antiviral response by targeting MAVS. Mol Immunol. (2018) 104:1–10. 10.1016/J.MOLIMM.2018.10.017 30390472

[329] Zhang W Wang G Xu Z-G Tu H Hu F Dai J. Lactate is a natural suppressor of RLR signaling by targeting MAVS. Cell. (2019) 178:176–189.e15. 10.1016/j.cell.2019.05.003 31155231 PMC6625351

[330] Cui J Zhu L Xia X Wang HY Legras X Hong J. NLRC5 negatively regulates the NF-kappaB and type I interferon signaling pathways. Cell. (2010) 141:483–96. 10.1016/j.cell.2010.03.040 20434986 PMC3150216

[331] Pattabhi S Knoll ML Gale M Loo Y-M. DHX15 is a coreceptor for RLR signaling that promotes antiviral defense against RNA virus infection. J Interf Cytokine Res. (2019) 39:331–46. 10.1089/jir.2018.0163 31090472 PMC6590726

[332] Yang Y-K Qu H Gao D Di W Chen H-W Guo X. ARF-like protein 16 (ARL16) inhibits RIG-I by binding with its C-terminal domain in a GTP-dependent manner. J Biol Chem. (2011) 286:10568–80. 10.1074/jbc.M110.206896 21233210 PMC3060509

[333] Kitai Y Takeuchi O Kawasaki T Ori D Sueyoshi T Murase M. Negative regulation of melanoma differentiation-associated gene 5 (MDA5)-dependent antiviral innate immune responses by Arf-like protein 5B. J Biol Chem. (2015) 290:1269–80. 10.1074/jbc.M114.611053 25451939 PMC4294491

[334] Ranjith-Kumar CT Lai Y Sarisky RT Kao CC. Green tea catechin, epigallocatechin gallate, suppresses signaling by the dsRNA innate immune receptor RIG-I. PLoS ONE. (2010) 5:1–11. 10.1371/journal.pone.0012878 20877565 PMC2943919

[335] Story RM Li H Abelson JN. Crystal structure of a DEAD box protein from the hyperthermophile Methanococcus jannaschii. Proc Natl Acad Sci USA. (2001) 98:1465–70. 10.1073/pnas.98.4.1465 11171974 PMC29280

[336] Heung LJ Del Poeta M. Unlocking the DEAD-box: a key to cryptococcal virulence? J Clin Invest. (2005) 115:593–5. 10.1172/JCI24508 15765144 PMC1052016

[337] Poynter S Lisser G Monjo A DeWitte-Orr S Poynter S Lisser G. Sensors of infection: viral nucleic acid PRRs in fish. Biology. (2015) 4:460–93. 10.3390/biology4030460 26184332 PMC4588145

[338] Barber MRW Aldridge JR Webster RG Magor KE Magor KE. Association of RIG-I with innate immunity of ducks to influenza. Proc Natl Acad Sci USA. (2010) 107:5913–8. 10.1073/pnas.1001755107 20308570 PMC2851864

[339] Rajendran KV Zhang J Liu S Peatman E Kucuktas H Wang X. Pathogen recognition receptors in channel catfish: II. Identification, phylogeny and expression of retinoic acid-inducible gene I (RIG-I)-like receptors (RLRs). Dev Comp Immunol. (2012) 37:381–9. 10.1016/J.DCI.2012.02.004 22387588

[340] Sun Y Ding N Ding SS Yu S Meng C Chen H. Goose RIG-I functions in innate immunity against Newcastle disease virus infections. Mol Immunol. (2013) 53:321–7. 10.1016/J.MOLIMM.2012.08.022 23063767

[341] Aoki T Hikima J Hwang SD Jung TS. Innate immunity of finfish: primordial conservation and function of viral RNA sensors in teleosts. Fish Shellfish Immunol. (2013) 35:1689–702. 10.1016/J.FSI.2013.02.005 23462146

[342] He Y Pan H Zhang G He S. Comparative study on pattern recognition receptors in non-teleost ray-finned fishes and their evolutionary significance in primitive vertebrates. Sci China Life Sci. (2019) 62:566–78. 10.1007/s11427-019-9481-8 30929190

[343] Chen J Shang S Wu X Zhong H Zhao C Wei Q. Genomic analysis and adaptive evolution of the RIG-I-like and NOD-like receptors in reptiles. Int J Biol Macromol. (2019) 134:1045–51. 10.1016/J.IJBIOMAC.2019.05.172 31129211

[344] Inohara N Chamaillard M McDonald C Nuñez G. NOD-LRR PROTEINS: role in host-microbial interactions and inflammatory disease. Annu Rev Biochem. (2005) 74:355–83. 10.1146/annurev.biochem.74.082803.133347 15952891

[345] Kersse K Vanden Berghe T Lamkanfi M Vandenabeele P. A phylogenetic and functional overview of inflammatory caspases and caspase-1-related CARD-only proteins. Biochem Soc Trans. (2007) 35:1508–11. 10.1042/BST0351508 18031255

[346] Kufer TA Fritz JH Philpott DJ. NACHT-LRR proteins (NLRs) in bacterial infection and immunity. Trends Microbiol. (2005) 13:381–8. 10.1016/j.tim.2005.06.004 15994078

[347] Chiu J DeSalle R Lam HM Meisel L Coruzzi G. Molecular evolution of glutamate receptors: a primitive signaling mechanism that existed before plants and animals diverged. Mol Biol Evol. (1999) 16:826–38. 10.1093/oxfordjournals.molbev.a026167 10368960

[348] Leszczyniecka M DeSalle R Kang D Fisher PB. The origin of polynucleotide phosphorylase domains. Mol Phylogenet Evol. (2004) 31:123–30. 10.1016/J.YMPEV.2003.07.012 15019613

[349] Cagliani R Forni D Tresoldi C Pozzoli U Filippi G Rainone V. RIG-I-like receptors evolved adaptively in mammals, with parallel evolution at LGP2 and RIG-I. J Mol Biol. (2014) 426:1351–65. 10.1016/j.jmb.2013.10.040 24211720

[350] Li N Li A Zheng K Liu X Gao L Liu D. Identification and characterization of an atypical RIG-I encoded by planarian *Dugesia japonica* and its essential role in the immune response. Dev Comp Immunol. (2019) 91:72–84. 10.1016/J.DCI.2018.10.007 30355517

[351] Coffman SR Lu J Guo X Zhong J Jiang H Broitman-Maduro G. Caenorhabditis elegans RIG-I homolog mediates antiviral RNA interference downstream of dicer-dependent biogenesis of viral small interfering RNAs. MBio. (2017) 8:e00264–17. 10.1128/mBio.00264-17 28325765 PMC5362034

[352] Wang P-H Weng S-P He J-G. Nucleic acid-induced antiviral immunity in invertebrates: an evolutionary perspective. Dev Comp Immunol. (2015) 48:291–6. 10.1016/J.DCI.2014.03.013 24685509

[353] Eyk CL Samaraweera SE Scott A Webber DL Harvey DP Mecinger O. ‘Non-self' mutation: double-stranded RNA elicits antiviral pathogenic response in a Drosophila model of expanded CAG repeat neurodegenerative diseases. Hum Mol Genet. (2019). 10.1093/hmg/ddz096. [Epub ahead of print]. 31071221

[354] Zipfel C. Plant pattern-recognition receptors. Trends Immunol. (2014) 35:345–51. 10.1016/j.it.2014.05.004 24946686

[355] Incarbone M Dunoyer P. RNA silencing and its suppression: novel insights from in planta analyses. Trends Plant Sci. (2013) 18:382–92. 10.1016/j.tplants.2013.04.001 23684690

[356] Kørner CJ Klauser D Niehl A Domínguez-Ferreras A Chinchilla D Boller T. The immunity regulator BAK1 contributes to resistance against diverse RNA viruses. Mol Plant-Microbe Interact. (2013) 26:1271–80. 10.1094/MPMI-06-13-0179-R 23902263

[357] Gong X-Y Zhang Q-M Gui J-F Zhang Y-B. SVCV infection triggers fish IFN response through RLR signaling pathway. Fish Shellfish Immunol. (2019) 86:1058–63. 10.1016/J.FSI.2018.12.063 30593899

[358] Zhang Q-M Zhao X Li Z Wu M Gui J-F Zhang Y-B. Alternative splicing transcripts of zebrafish LGP2 gene differentially contribute to IFN antiviral response. J Immunol. (2018) 200:688–703. 10.4049/jimmunol.1701388 29203516

[359] Xu T Chu Q Cui J Bi D. Inducible MicroRNA-3570 feedback inhibits the RIG-I-dependent innate immune response to rhabdovirus in teleost fish by targeting MAVS/IPS-1. J Virol. (2018) 92:e01594–17. 10.1128/JVI.01594-17 29093090 PMC5752954

[360] Sun Y Han J Chu Q Liu X Xu T. microRNA-210 participates in regulating RIG-I signaling pathway via targeting DUBA in miiuy croaker after poly(I:C) stimulation. Fish Shellfish Immunol. (2018) 77:1–7. 10.1016/J.FSI.2018.02.003 29408541

[361] Gu T Lu L An C Chen B Wei W Wu X. MDA5 and LGP2 acts as a key regulator though activating NF-κB and IRF3 in RLRs signaling of mandarinfish. Fish Shellfish Immunol. (2019) 86:1114–22. 10.1016/J.FSI.2018.12.054 30594581

[362] Zou PF Chang MX Li Y Huan Zhang S Fu JP Chen SN. Higher antiviral response of RIG-I through enhancing RIG-I/MAVS-mediated signaling by its long insertion variant in zebrafish. Fish Shellfish Immunol. (2015) 43:13–24. 10.1016/J.FSI.2014.12.001 25524497

[363] Gack MU Kirchhofer A Shin YC Inn K-S Liang C Cui S. Roles of RIG-I N-terminal tandem CARD and splice variant in TRIM25-mediated antiviral signal transduction. Proc Natl Acad Sci USA. (2008) 105:16743–8. 10.1073/pnas.0804947105 18948594 PMC2575490

[364] Miranzo-Navarro D Magor KE. Activation of duck RIG-I by TRIM25 is independent of anchored ubiquitin. PLoS ONE. (2014) 9:e86968. 10.1371/journal.pone.0086968 24466302 PMC3900705

[365] Helin AS Wille M Atterby C Järhult J Waldenström J Chapman JR. Expression of immune genes RIG-I and Mx in mallard ducks infected with low pathogenic avian influenza (LPAI): a dataset. Data Br. (2018) 18:1562–6. 10.1016/j.dib.2018.04.061 29904657 PMC5998173

[366] Huo H Wang Y Wang D Wang Y Chen X Zhao L. Duck RIG-I restricts duck enteritis virus infection. Vet Microbiol. (2019) 230:78–85. 10.1016/J.VETMIC.2019.01.014 30827409

[367] Sun X Li W Liu E Huang H Wang T Wang X. *In vivo* cellular and molecular study on duck spleen infected by duck Tembusu virus. Vet Microbiol. (2019) 230:32–44. 10.1016/J.VETMIC.2018.12.003 30827402

[368] Wei LM Jiao PR Song YF Han F Cao L Yang F. Identification and expression profiling analysis of goose melanoma differentiation associated gene 5 (MDA5) gene. Poult Sci. (2013) 92:2618–24. 10.3382/ps.2013-03064 24046408

[369] Sun Y Mao X Zheng H Wu W Rehman ZU Liao Y. Goose MAVS functions in RIG-I-mediated IFN-β signaling activation. Dev Comp Immunol. (2019) 93:58–65. 10.1016/J.DCI.2018.12.006 30557581

[370] Karpala AJ Stewart C McKay J Lowenthal JW Bean AGD. Characterization of chicken Mda5 activity: regulation of IFN-β in the absence of RIG-I functionality. J Immunol. (2011) 186:5397–405. 10.4049/jimmunol.1003712 21444763

[371] Liniger M Summerfield A Zimmer G McCullough KC Ruggli N. Chicken cells sense influenza A virus infection through MDA5 and CARDIF signaling involving LGP2. J Virol. (2012) 86:705. 10.1128/JVI.00742-11 22072756 PMC3255855

[372] Hayashi T Watanabe C Suzuki Y Tanikawa T Uchida Y Saito T. Chicken MDA5 senses short double-stranded RNA with implications for antiviral response against avian influenza viruses in chicken. J Innate Immun. (2014) 6:58–71. 10.1159/000351583 23860388 PMC6741575

[373] Li G-Q Tian Y Chen L Shen J-D Tao Z-R Zeng T. Cloning, expression and bioinformatics analysis of a putative pigeon melanoma differentiation-associated gene 5. Br Poult Sci. (2018) 60:94–104. 10.1080/00071668.2018.1564241 30595037

[374] Xu L Yu D Fan Y Peng L Wu Y Yao Y-G. Loss of RIG-I leads to a functional replacement with MDA5 in the Chinese tree shrew. Proc Natl Acad Sci USA. (2016) 113:10950–5. 10.1073/pnas.1604939113 27621475 PMC5047190

[375] Wei Y Zhou H Wang A Sun L Wang M Jia R. TRIM25 identification in the Chinese goose: gene structure, tissue expression profiles, and antiviral immune responses *in vivo* and *in vitro*. Biomed Res Int. (2016) 2016:1403984. 10.1155/2016/1403984 27995135 PMC5138445

[376] Chang M Collet B Nie P Lester K Campbell S Secombes CJ. Expression and functional characterization of the RIG-I-like receptors MDA5 and LGP2 in Rainbow trout (*Oncorhynchus mykiss*). J Virol. (2011) 85:8403–12. 10.1128/JVI.00445-10 21680521 PMC3147945

[377] Weber M Sediri H Felgenhauer U Binzen I Bänfer S Jacob R. Influenza virus adaptation PB2-627K modulates nucleocapsid inhibition by the pathogen sensor RIG-I. Cell Host Microbe. (2015) 17:309–19. 10.1016/J.CHOM.2015.01.005 25704008 PMC4359673

[378] Ariumi Y. Multiple functions of DDX3 RNA helicase in gene regulation, tumorigenesis, and viral infection. Front Genet. (2014) 5:423. 10.3389/fgene.2014.00423 25538732 PMC4257086

[379] Valiente-Echeverría F Hermoso MA Soto-Rifo R. RNA helicase DDX3: at the crossroad of viral replication and antiviral immunity. Rev Med Virol. (2015) 25:286–99. 10.1002/rmv.1845 26174373

[380] Gringhuis SI Hertoghs N Kaptein TM Zijlstra-Willems EM Sarrami-Fooroshani R Sprokholt JK HIV-1 blocks the signaling adaptor MAVS to evade antiviral host defense after sensing of abortive HIV-1 RNA by the host helicase DDX3. Nat Immunol. (2016) 18:225–35. 10.1038/ni.3647 28024153

[381] Szappanos D Tschismarov R Perlot T Westermayer S Fischer K Platanitis E. The RNA helicase DDX3X is an essential mediator of innate antimicrobial immunity. PLOS Pathog. (2018) 14:e1007397. 10.1371/journal.ppat.1007397 30475900 PMC6283616

[382] Zhang Z Yuan B Lu N Facchinetti V Liu Y-J. DHX9 pairs with IPS-1 to sense double-stranded RNA in myeloid dendritic cells. J Immunol. (2011) 187:4501–8. 10.4049/jimmunol.1101307 21957149 PMC3656476

[383] Lu H Lu N Weng L Yuan B Liu Y-J Zhang Z. DHX15 senses double-stranded RNA in myeloid dendritic cells. J Immunol. (2014) 193:1364–72. 10.4049/jimmunol.1303322 24990078 PMC4108507

[384] Zhang Z Kim T Bao M Facchinetti V Jung SY Ghaffari AA. DDX1, DDX21, and DHX36 helicases form a complex with the adaptor molecule TRIF to sense dsRNA in dendritic cells. Immunity. (2011) 34:866–78. 10.1016/j.immuni.2011.03.027 21703541 PMC3652560

[385] Liu Y Lu N Yuan B Weng L Wang F Liu Y-J. The interaction between the helicase DHX33 and IPS-1 as a novel pathway to sense double-stranded RNA and RNA viruses in myeloid dendritic cells. Cell Mol Immunol. (2014) 11:49–57. 10.1038/cmi.2013.40 24037184 PMC4002151

[386] Zhang K Zhang Y Xue J Meng Q Liu H Bi C. DDX19 inhibits type I interferon production by disrupting TBK1-IKKε-IRF3 interactions and promoting TBK1 and IKKε degradation. Cell Rep. (2019) 26:1258–72.e4. 10.1016/j.celrep.2019.01.029 30699353

[387] Loureiro ME Zorzetto-Fernandes AL Radoshitzky S Chi X Dallari S Marooki N. DDX3 suppresses type I interferons and favors viral replication during Arenavirus infection. PLOS Pathog. (2018) 14:e1007125. 10.1371/journal.ppat.1007125 30001425 PMC6042795

[388] Park E Byun YH Park S Jang YH Han W Won J. Co-degradation of interferon signaling factor DDX3 by PB1-F2 as a basis for high virulence of 1918 pandemic influenza. EMBO J. (2019) 38:e99475. 10.15252/embj.201899475 30979777 PMC6518015

[389] Mosallanejad K Sekine Y Ishikura-Kinoshita S Kumagai K Nagano T Matsuzawa A. The DEAH-box RNA helicase DHX15 activates NF-κB and MAPK signaling downstream of MAVS during antiviral responses. Sci Signal. (2014) 7:ra40. 10.1126/scisignal.2004841 24782566

[390] Miyashita M Oshiumi H Matsumoto M Seya T. DDX60, a DEXD/H box helicase, is a novel antiviral factor promoting RIG-I-like receptor-mediated signaling. Mol Cell Biol. (2011) 31:3802–19. 10.1128/MCB.01368-10 21791617 PMC3165724

[391] Oshiumi H Miyashita M Okamoto M Morioka Y Okabe M Matsumoto M. DDX60 is involved in RIG-I-dependent and independent antiviral responses, and its function is attenuated by virus-induced EGFR activation. Cell Rep. (2015) 11:1193–207. 10.1016/j.celrep.2015.04.047 25981042

[392] Zhu Q Tan P Li Y Lin M Li C Mao J. DHX29 functions as an RNA co-sensor for MDA5-mediated EMCV-specific antiviral immunity. PLoS Pathog. (2018) 14:e1006886. 10.1371/journal.ppat.1006886 29462185 PMC5834211

[393] Elion DL Cook RS. Activation of RIG-I signaling to increase the pro-inflammatory phenotype of a tumor. Oncotarget. (2019) 10:2338–9. 10.18632/oncotarget.26729 31040925 PMC6481327

[394] Aznar MA Planelles L Perez-Olivares M Molina C Garasa S Etxeberría I. Immunotherapeutic effects of intratumoral nanoplexed poly I:C. J Immunother Cancer. (2019) 7:116. 10.1186/s40425-019-0568-2 31046839 PMC6498680

[395] Yong HY Luo D. RIG-I-like receptors as novel targets for pan-antivirals and vaccine adjuvants against emerging and re-emerging viral infections. Front Immunol. (2018) 9:1379. 10.3389/fimmu.2018.01379 29973930 PMC6019452

